# A methodological systematic review of meta-ethnography conduct to articulate the complex analytical phases

**DOI:** 10.1186/s12874-019-0670-7

**Published:** 2019-02-18

**Authors:** Emma F. France, Isabelle Uny, Nicola Ring, Ruth L. Turley, Margaret Maxwell, Edward A. S. Duncan, Ruth G. Jepson, Rachel J. Roberts, Jane Noyes

**Affiliations:** 1 NMAHP Research Unit, University of Stirling, Unit 13 Scion House, Stirling University Innovation Park, Stirling, FK9 4NF UK; 2 School of Health and Social Care, Edinburgh Napier University, Sighthill Campus, Sighthill Court, Edinburgh, EH11 4BN UK; 3 DECIPHEr, School of Social Sciences, Cardiff University, Glamorgan Building, King Edward VII, Cardiff, CF10 3WT UK; 4 Scottish Collaboration for Public Health Research and Policy, University of Edinburgh, 20 West Richmond Street, Edinburgh, EH8 9DX UK; 5 School of Health Sciences, Bangor University, Bangor, Gwynedd LL57 2EF UK

**Keywords:** Meta-ethnography, Systematic review, Qualitative evidence synthesis, Meta-synthesis, Qualitative research, Research design, Methodology

## Abstract

**Background:**

Decision making in health and social care requires robust syntheses of both quantitative and qualitative evidence. Meta-ethnography is a seven-phase methodology for synthesising qualitative studies. Developed in 1988 by sociologists in education Noblit and Hare, meta-ethnography has evolved since its inception; it is now widely used in healthcare research and is gaining popularity in education research. The aim of this article is to provide up-to-date, in-depth guidance on conducting the complex analytic synthesis phases 4 to 6 of meta-ethnography through analysis of the latest methodological evidence.

**Methods:**

We report findings from a methodological systematic review conducted from 2015 to 2016. Fourteen databases and five other online resources were searched. Expansive searches were also conducted resulting in inclusion of 57 publications on meta-ethnography conduct and reporting from a range of academic disciplines published from 1988 to 2016.

**Results:**

Current guidance on applying meta-ethnography originates from a small group of researchers using the methodology in a health context. We identified that researchers have operationalised the analysis and synthesis methods of meta-ethnography – determining how studies are related (phase 4), translating studies into one another (phase 5), synthesising translations (phase 6) and line of argument synthesis - to suit their own syntheses resulting in variation in methods and their application. Empirical research is required to compare the impact of different methods of translation and synthesis. Some methods are potentially better at preserving links with the context and meaning of primary studies, a key principle of meta-ethnography. A meta-ethnography can and should include reciprocal and refutational translation and line of argument synthesis, rather than only one of these, to maximise the impact of its outputs.

**Conclusion:**

The current work is the first to articulate and differentiate the methodological variations and their application for different purposes and represents a significant advance in the understanding of the methodological application of meta-ethnography.

**Electronic supplementary material:**

The online version of this article (10.1186/s12874-019-0670-7) contains supplementary material, which is available to authorized users.

## Background

Synthesising multiple qualitative primary research studies, referred to as ‘qualitative evidence synthesis’ by the Cochrane Qualitative Methods Implementation Group, is increasingly gaining acceptance as a valid and rigorous way to distil qualitative evidence to inform health and social care decision making [1–8]. Noblit and Hare’s [9] meta-ethnography, originally developed for synthesising education ethnographies, is one of the most frequently used and influential methodologies for qualitative evidence synthesis in health and social care research [10–12] with a rapidly increasing volume of published meta-ethnographies [10, 12, 13].

Meta-ethnography is theoretically-based drawing on Geertz’s concept of thick description [14] and Turner’s [15] theory of sociological understanding as ‘translation.’ It is unique among qualitative evidence synthesis methodologies in synthesising conceptual data from primary studies and was designed specifically to take into account the unique research contexts in primary studies. Meta-ethnography is suited to producing a new interpretation, model or theory, which goes beyond the findings of the individual studies synthesised, and does not simply aggregate findings [9]. In our view, theory development is one of meta-ethnography’s key strengths. If adequately conducted and reported, meta-ethnography has the potential to generate new evidence on how patients experience their own health condition, disease, or treatments and how this may influence their adherence to treatments [1]. It may also help us to understand why interventions or services work in certain settings but not in others [16]. For instance, meta-ethnographies have been included in clinical guidelines for asthma management [17, 18], medication adherence [4, 5] and head and neck cancer care [7, 8].

Meta-ethnography has seven iterative and overlapping phases [9], which we now describe with emphasis on the complex analytic synthesis Phases 4 to 6.

### Phase 1 Getting started

This involves deciding the focus of the synthesis. Noblit and Hare [9] described this phase as ‘identifying an intellectual interest that qualitative research might inform’ ([9], pp. 26–27).

### Phase 2 Deciding what is relevant to the initial interest

This comprises identifying and selecting study accounts to synthesise. Noblit and Hare did not advise in detail how to do this. Unlike many recent health-related meta-ethnographies [10, 19] they selected studies purposefully; they did not employ systematic review methods, which had not been developed in the 1980s.

### Phase 3 Reading the studies

Noblit and Hare [9] described this phase as ‘the repeated reading of the accounts and the noting of interpretative metaphors [...] this requires extensive attention to the details in the accounts.’ ([9], p. 28).

### Phase 4 Determining how the studies are related

Noblit and Hare [9] described the process of Phase 4 as:

‘the various studies must be “put together.” This requires determining the relationships between the studies to be synthesized. We think it makes sense to create a list of the key metaphors, phrases, ideas, and/ or concepts (and their relations) used in each account and to juxtapose them.’ ([9], p. 28).

Noblit and Hare [9] stated that when deciding how studies relate reviewers should consider what the studies are about, the theoretical approach of studies, and the meaning of their concepts, themes or metaphors. They explained three different ways in which studies might be related:

1. the accounts are directly comparable as ‘reciprocal translations’
2. the accounts stand in relative opposition to each other and are essentially ‘refutational,’ or
3. the ‘dissimilar but related studies’ ([9], p. 64) taken together represent a ‘line of argument,’ also described as a process of discovering ‘a “whole” among a set of parts’. ([9], p. 63).

Noblit and Hare called Phase 4 a ‘key judgment call’ ([9], p. 81) because reviewers must determine the relationship between studies in order to decide how to synthesise them.

### Phase 5 Translating the studies into one another

This is one level of synthesis involving systematically comparing the meaning of metaphors, concepts or themes and their relations across study accounts to identify the range of metaphors, concepts, and themes. Translation is underpinned by the theory of social explanation [15] and also draws on Brown’s (1977 in [9]) idea that all knowledge is metaphoric. Noblit and Hare said:

‘we have adapted Turner’s (1980) notion that all explanation is essentially comparative and takes the form of translation. A meta-ethnography based in Turner’s conceptualization simply extends his argument by constructing syntheses by translating multiple qualitative studies into one another’s terms’ ([9], p. 25).

Interpreting *meaning* is key to translation, i.e. translation is idiomatic rather than literal, and should take account of each study’s context (e.g. where and when it was done and with whom) [9]. There are two types of translation described by Noblit and Hare [9]: reciprocal translation and refutational translation. They specified that reciprocal translation.

‘requires the assumption that the studies can be “added” together. That is, they are clearly studies about some similar things’ ([9], p. 40).

They described the conduct of reciprocal translation as follows:

‘we conceive of meta-ethnographic syntheses as translations (one case is like another, except that . . .). […] in an iterative fashion, each study is translated into terms (metaphors) of the others and vice versa.’ ([9], p. 38).

When the studies are not similar enough to be added together then it may be appropriate to conduct refutational translation, which Noblit and Hare [9] described as a particular type of interpretation:

‘Ethnographies that are implicitly or explicitly refutations of each other […] require a more elaborate set of translations - translations of both the ethnographic accounts and the refutations […]. Our approach treats the refutation itself as part of the interpretation to be synthesized.’ ([9], pp. 47-48).

Attention should be paid to the assumptions, motivations and ideology behind a study. A benefit of conducting a refutational translation is that it allows reviewers to identify if the theories or ideologies underlying two or more studies differ [9].

### Phase 6 Synthesising translations

Noblit and Hare [9] defined phase 6 as follows:

‘Synthesis refers to making a whole into something more than the parts alone imply. [..] when the number of studies is large and the resultant translations numerous, the various translations can be compared with one another to determine if there are types of translations or if some metaphors and/or concepts are able to encompass those of other accounts. ([9], p. 29).’

This is a process of going beyond the findings of any individual study. It is ‘a second level of synthesis’ ([9], p. 28) in which the translations from phase 5 are compared to identify common or overarching concepts and to develop new interpretations from these.

### Phase 7 Expressing the synthesis

Communicating the synthesis to your audience in a suitable format [9].

Translation, synthesising translations and line of argument synthesis are particularly poorly understood, as evidenced by many published meta-ethnographies which state that they have used meta-ethnography when they have not adhered to the principles of the methdolology [10, 12]. These complex synthesis processes form the heart of the methodology, but were not described in detail by Noblit and Hare [9] whose seminal publication was not intended to be a step-by-step, procedural guide. Indeed, Noblit (in Thorne et al. [20]) has stated that the 1988 book was not a definitive work on meta-ethnography, hence there are gaps. Meta-ethnography has evolved since 1988 (e.g. [1, 21]), mainly as a result of its application and adaption in health research [13], but there is little guidance on its conduct which incorporates recent methodological developments. Poor meta-ethnography conduct and reporting may limit the effective use of meta-ethnography findings [3, 10] and indicate the need for more detailed and current guidance on conduct.

The findings reported in this article come from a methodological systematic review to identify recommendations and guidance for conducting and reporting a meta-ethnography carried out as part of the eMERGe project [22] which created the first methodology-specific reporting guidance for meta-ethnography [23–27]. The aim of this article is to give guidance on the conduct of the complex analytical stages – phases 4 to 6 – of meta-ethnography through analysis of the latest methodological evidence identified from publications included in our systematic review. Specifically, we describe, contrast and critique different methods for conducting phases 4 to 6.

## Methods

Data for this article were drawn from the systematic review (PROSPERO CRD42015024709), for which we now describe the methods [22, 23]. The review question was: what are the existing recommendations and guidance for conducting and reporting each process in a meta-ethnography, and why?

### Literature search methods

A comprehensive search for published and unpublished texts in any language was performed in multiple information sources. Fourteen bibliographic databases and five other electronic resources covering a spectrum of academic disciplines were searched between June and August 2015. The search strategy was first designed in Medline following testing and refining against a set of key papers and then adapted to the remaining databases (listed in Additional file 1). An example of the search terms used in the review, based on those used for Medline, is:

1. (“qualitative synthes#s” or Qualitative systematic review*).ti,ab.
2. (“meta-ethnograph*” or “metaethnograph*” or “meta ethnograph*” or “meta-synth*” or “meta synth*” or “metasynth*” or “line* of argument”).ti,ab.
3. (“critical synth*” or “textual synth*” or “framework synth*” or “thematic synth*” or “grounded synth*” or textual narrative synthe#s) adj2 review*).ti,ab.
4. (“metasynthes#s” or “meta synthes#s” or “metasynthes#s” or “meta-stud*” or metastud*).ti,ab.
5. ((“qualitative” adj2 “synth*”) or (“third order” adj2 “construct*”) or (qualitative adj2 review)).ti,ab.
6. knowledge synthesis.ti,ab.
7. or/1–6
8. ((“method*” or steps) adj2 (“insight*” or lessons or learnt or “explor*” or learned or conduct* or “approach*”)).ti,ab.
9. “worked example*”.ti,ab.
10. ((good or best or recommend* or quality or publishing or reporting) adj3 (guid* or design* or standard* or practi#e* or report* or method* or steps)).ti,ab.
11. lessons learnt.ti,ab.
12. ((challenges or steps) adj5 (synthesis* or qualitative or conduct* or report* or design* or method* or present* or practical*)).ti,ab.
13. (practical adj5 (guid* or design* or standard* or approach* or framework*)).ti,ab.
14. ((methods or methodological) adj5 (guid* or design* or standard* or approach* or framework*)).ti,ab.
15. **or/8–14**
16. qualitative research/ and “meta-analysis as topic”/
17. **15 and 7**
18. **16 or 17**

We also employed expansive search techniques which involved gathering relevant publications known to our expert panel and the project team; forward and backward citation tracking of all included publications (i.e. checking if there were any further relevant texts that either cited or were cited by included publications), and citation alerts. Any new relevant published or in press publications identified through these expansive methods were included up to March 2016.

#### Screening and selection

Literature search results were downloaded into Endnote® bibliographic software and screened against eligibility criteria presented in Table 1. Titles and abstracts were first screened independently by two reviewers for references published from 2006 onwards (6271 records) and by one reviewer for references published before 2006 (1251 records), owing to time and resource constraints. Based on our familiarity with the literature, we were confident that references prior to 2006 were known to the project team and its expert advisors already, or they would be identified through expansive searches. Any publications identified as potentially relevant were then retrieved in full-text and screened by two independent reviewers, with any disagreements resolved through discussion or by a third reviewer.

**Table 1:** Systematic review inclusion and exclusion criteria

Exclusion criteria	Inclusion criteria
1. Does not report on methodological issues^a^ in conducting meta-ethnography AND 2. is not a reporting guideline/ providing guidance on reporting meta-ethnography	1. Reports on methodological issues^a^ in conducting meta-ethnography OR 2. Is a reporting guideline for or provides guidance on reporting qualitative syntheses including meta-ethnography
3. Published before 1988 (date of the publication of the original meta-ethnography text by Noblit and Hare)	3. Published after 1988
4. Theses below PhD level	4. Book, book chapter, journal article/ editorial, report or PhD thesis
	5. Any language
	6. Any discipline or topic (not just health related)

^a^‘Methodological issues’ included all aspects of the meta-ethnography approach including: the philosophical and theoretical underpinnings; research design and the research practices and procedures including conveying findings and developing theory; also included, providing advice on initially choosing meta-ethnography as suitable for one’s research aim, defining the characteristics of a meta-ethnography, comparing qualitative synthesis methodologies including meta-ethnography as one of those compared, and/or describing in detail any other methodological aspect of meta-ethnography

### Data coding and analysis

Fifty-seven full texts were coded using qualitative analysis software NVivo 10.0 by four reviewers who used a bespoke coders’ guidance document developed, piloted, and refined by the team. One reviewer coded each publication; a second reviewer checked completeness of coding for 13 (23%) publications, judged as rich and/or seminal by the team, which confirmed that overall the coding guidance had been applied consistently and coding was accurate. ‘Nodes’ or coding categories were primarily based on the seven phases of meta-ethnography [9], with additional nodes for other relevant data (e.g. ‘definition or nature of meta-ethnography,’ ‘selection of a qualitative evidence synthesis approach’). Findings presented in this article focus on the conduct, not reporting, of meta-ethnography.

Full publications and coded data were read repeatedly and compared using constant comparison, mainly by two team members who recorded their analysis in memos in NVivo for each node. For nodes concerning the complex Phases 4, 5 and 6, each researcher independently identified the key themes and issues, then compared them and wrote a joint analytic memo. Each researcher maintained an analysis journal in NVivo to record development of ideas, and analysis decisions made at wider project team meetings were documented. Each researcher noted which publications they considered “rich in detail” about meta-ethnography overall and for phases 4 to 6, i.e. a detailed account with in-depth explanation and rationales that went beyond description. We wrote a detailed definition for each phase of a meta-ethnography, analysed and summarised advice and recommendations, and documented pitfalls in the conduct and reporting of meta-ethnography, noting any contradictions or uncertainties.

From our inclusion criteria, we developed a system to classify the publications according to the type of evidence they contributed to the review; where possible we differentiated between those based on the authors’ opinion and those which were supported by ‘evidence.’ Evidence could be empirical data from published literature or experience conducting a meta-ethnography, or reasoned argument. We developed seven main categories:

- A meta-ethnography with methodological detail on the application of methods (referred to as ‘worked examples’)
- Other methodological text (i.e. not a meta-ethnography) exploring particular aspects of meta-ethnography conduct in-depth (e.g. conduct of reciprocal translation)
- Critique of meta-ethnography
- Descriptive overview of the methodology (some of which compared qualitative evidence synthesis methodologies)
- Guidance on meta-ethnography conduct
- Reporting of meta-ethnography methods
- Generic reporting guideline for qualitative evidence syntheses that could potentially be applied to meta-ethnography.

To add rigour to the process and enhance interpretation, the preliminary review findings were presented to academic experts and other key stakeholders at various fora including:

- a project team meeting
- an online workshop in May 2016 with 12 academic experts in meta-ethnography, 3 professional end users of evidence syntheses, 11 lay people, and 5 project team members. A further six academics and three lay people commented on the workshop materials and notes after the workshop;
- a project advisory group meeting in November 2016 attended by 9 project team members, 1 independent chairperson, 7 lay advisors and 10 academic experts; and
- two formal and several informal meetings with one of the two originators of meta-ethnography, Professor George W. Noblit in June 2016.

These meetings added to our understanding of meta-ethnography conduct and have influenced the review findings; where a direct link can be traced from our findings to our discussions with stakeholders we state this. We describe the literature search and screening results, the characteristics of included publications, highlight the key findings and then focus in detail on the complex analytic synthesis Phases 4 to 6 which are often poorly understood and reported in published meta-ethnographies [10].

## Results

### Literature search and screening results

**Fig. 1:**
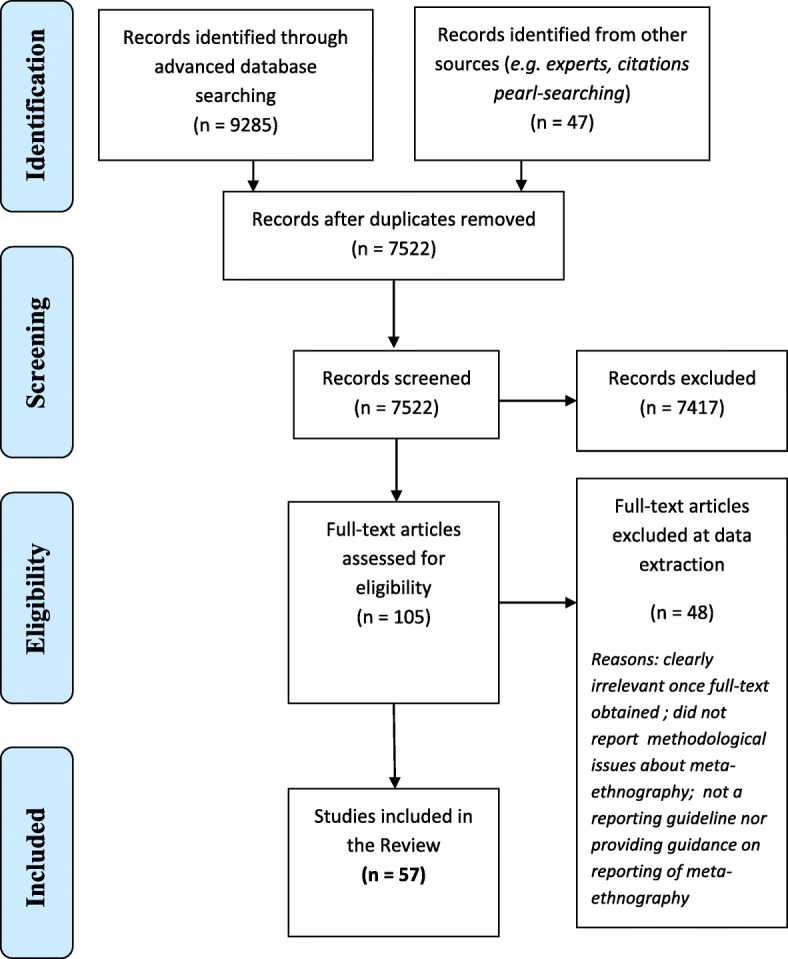
PRISMA flow diagram. Copyright statement: this PRISMA diagram contains public sector information licensed under the Open Government Licence v3.0. Adapted From: Moher D, Liberati A, Tetzlaff J, Altman DG, The PRISMA Group (2009). Preferred Reporting Items for Systematic Reviews and Meta-Analyses: The PRISMA Statement. PLoS Med 6(6): e1000097. doi:10.1371/journal.pmed1000097

Figure 1 presents the results of the literature searching and screening. The search returned 7522 references. 105 potentially relevant references were screened in full-text and 57 met our inclusion criteria.

### Study characteristics

Of the 57 publications, 56 were published from 2002 to 2016 with most (*N* = 37) being published from 2009 onwards. The majority (*N* = 44) were peer-reviewed journal articles and the remainder were books, book sections, PhD theses or reports. Forty-six publications came from health disciplines and 12 from non-health disciplines, mostly education and social work. Twenty-eight publications had solely UK-based authors. Nine publications contributed no data on phases 4 to 6 [28–36].

According to our classification of the publications, we identified 16 worked example meta-ethnographies with methodological detail on the application of methods; 12 other methodological texts exploring particular aspects of meta-ethnography conduct in-depth; five critiques of the methodology based on empirical data, e.g. from a systematic review, and two based on author opinion; 14 descriptive overviews of the methodology; three providing guidance on meta-ethnography conduct based on data or on opinion; and five with a focus on meta-ethnography reporting including a generic reporting guideline for qualitative evidence syntheses [30]. Figure 1 shows the screening results and Additional file 2: Table S2 shows the publication characteristics and whether they contributed data on phases 4 to 6. Fifteen publications were considered to contribute rich data on conduct of at least one of Phases 4 to 6 and five publications [1, 19, 37–39] contributed rich data for all three phases 4 to 6 (see Additional file 2: Table S2 and Additional file 3: Table S3). Those not contributing rich data usually provided only a sentence or two on conduct of Phases 4 to 6, often citing or paraphrasing Noblit and Hare [9] (e.g. [40–45], gave a brief one to two paragraph summary of conduct (e.g. [46]) and/or they focused more on reporting of meta-ethnography (e.g. [47]) – some examples of rich and not rich data are given in Additional file 3: Table S3. During analysis it became apparent that one publication [48] did not differentiate between meta-ethnography and other qualitative evidence synthesis methodologies, therefore it has been excluded from the findings.

### Findings from analysis of review publications

In our analysis of the content of the review publications we highlight that there is a distinct lack of empirical research comparing methods for conducting phases 4 to 6. We demonstrate that, since publication of Noblit and Hare’s [9] seminal book, researchers have interpreted and tailored the analytic synthesis methods of meta-ethnography to their purposes; several methods for reciprocal translation have evolved to deal with and suit different volumes and heterogeneity of data. Some of these translation methods appear to be truer than others to the original methodology. We also emphasise that a meta-ethnography is suited to synthesising rich data; should combine reciprocal, refutational and line of argument syntheses rather than being an either/or choice; and that synthesising translations and theory development, which are often not carried out in published meta-ethnographies [10], are a key part of meta-ethnography.

### Phase 4. “Determining How the Studies are Related”

Data from 25 publications (shown in Additional file 2: Table S2) were coded at the node for Phase 4, most did not provide rich detail on how to conduct Phase 4, however, seven worked examples of meta-ethnographies [1, 19, 37–39, 49, 50], all health-focused, gave a detailed description. Four of these publications [1, 38, 39, 49] are related, involving the same team.

Our stance is that translation does not have to be either reciprocal or refutational but that a meta-ethnography should involve both kinds of translation; this view was echoed by our stakeholders. Moreover, line of argument is not an alternative to conducting translation but in addition to it and a next step on from synthesising translations. We explore these issues further in the sections on Phases 5 and 6.

From our analysis of all relevant review publications, particularly the worked examples of meta-ethnographies, we identified that, closely following Noblit and Hare [9], reviewers operationalised Phase 4 as having three key steps:

1. listing, or otherwise documenting, data (concepts, themes, metaphors, findings) and how they relate to each other within each study account,
2. juxtaposing or comparing the data across studies,
3. using those data to determine the relationship between studies.

We now examine each step.

#### Listing concepts, themes, metaphors

The kinds of data from study accounts that were listed, recorded or extracted varied. Authors of one publication recorded only clearly-articulated ‘second order constructs’ (this term was not used by Noblit and Hare [9], it means the original study authors’ interpretation of participants’ views, expressed as themes and concepts) [19], others also included descriptive findings or themes [1, 37, 38, 49]. A concept can be defined as having ‘some analytic or conceptual power, unlike more descriptive themes’ ([38], p. 46). Furthermore, some authors extracted ‘first order constructs’ (research participants’ quotations) as well as second order constructs (the original authors’ concepts) [37, 38]. However, the distinction between first and second order constructs is not clear-cut because authors select participant quotes to support their second order constructs [19]. For this reason, we believe that first order constructs can be analysed and synthesised along with their corresponding second order constructs but not in isolation. Analysing descriptive data can be problematic because the reviewers first have to interpret it without fully understanding or having access to the context of the primary studies [19, 51]. Findings reported in qualitative health-related journal articles are often descriptive [21] but are not usually rich (highly detailed) descriptions, such as in an ethnographic monograph of the kind Noblit and Hare [9] were synthesising. Therefore, we suggest that analysing studies containing conceptual data, or rich description, is a key part of a meta-ethnography; this view was influenced by discussions with our stakeholders including George Noblit. Reviewers should consider whether to exclude ‘thin’ (i.e. superficial) descriptive accounts. Once conceptual data from study accounts had been recorded, authors usually explored the relationship between studies.

#### Methods for listing and juxtaposing concepts, themes, metaphors

Noblit and Hare [9] gave examples of how they listed metaphors in their meta-ethnographies, but did not give detailed guidance. Authors of the worked examples of meta-ethnographies used different, but comparable, processes to operationalise the listing and juxtaposing of concept/metaphors in Phase 4 including: lists [1, 44, 45, 49], diagrams [1, 37, 49], tables [37, 39, 50–52], and coding using qualitative analysis software [19]. Campbell et al. [1, 49] created hand-written lists of summarised concepts and findings from studies and then drew lines and arrows between related concepts in the various studies. Pope [44] suggested that reviewers compile a list of ideas, key concepts, and explanatory schema. Another approach was to create a table or grid laid out to display commonalities and differences between concepts [37, 39, 50] and sometimes the relationships between concepts within each study and between studies [50]. The table might also contain important contextual data about each study, such as its setting [39] and the reviewers’ preliminary interpretations [37]. In addition to a table, Malpass et al. [37] drew conceptual diagrams for each paper to record relations between the concepts within studies. A unique approach was to use qualitative analysis software NVivo 9 to record concepts [19]. This involved a team of reviewers independently coding conceptual findings from anywhere in the study accounts. More detail is given in the illustrative case studies. These different methods for conducting the various processes in Phase 4 have not been compared empirically, however, a table or list would be unwieldy for hundreds of concepts, hand writing lists is labour-intensive, and coding in NVivo is efficient (and streamlines many of the processes in Phases 3 to 6) but might make recording links between concepts within primary study accounts more difficult.

#### Methods for determining how studies were related

Deciding how the studies relate is a process somewhat idiosyncratic to the specific meta-ethnography, partly determined by its aim and focus. We identified two main approaches related to two out of three possible methods (focus, theory, meaning of concepts) suggested by Noblit and Hare [9]:

- grouping studies by their focus (what the studies are about) [1, 37, 38, 49] and
- grouping common concepts from studies (i.e. by the meaning of their concepts, themes or metaphors) [21, 39, 50].

We did not observe worked examples relating studies by their theoretical approach either because this activity was not carried out, or it tended to be done during other phases, e.g. phases 1 and 2 (e.g. [50]). We identified that other aspects of studies also can be compared to explore the relationship between them, e.g., the research design, research participant characteristics, and other aspects of context, such as when and where they were conducted [24].

### Grouping study accounts by their focus

One approach to relating studies was to group accounts by their conceptual focus relating to the topic, e.g. patients’ experience of antidepressants [37] or type of medicine [1, 38], and to then synthesise each of the groups separately before synthesising across the groups [1, 37, 49]. This was done to deal with heterogeneity of studies (in terms of their focus) and a large volume of accounts [1].

### Grouping common concepts from studies

Another approach used to deal with a large volume of data [19] and/or heterogenous data [21, 50] was to group concepts as opposed to study accounts, for instance, by conducting a form of thematic analysis [21], gathering similar themes from studies into ‘piles’ or categories of shared meaning [19], or organising concepts from studies according to common underlying metaphors [50]. This approach was observed in three worked examples [19, 21, 50], for example, Atkins et al. [21] had a large volume of heterogeneous concepts, whereas Erasmus [50] had a small volume of accounts (*N* = 4) with heterogeneous focuses.

#### Insights on phase 4

We observed that the process of relating studies starts during Phases 1 and 2: a tightly-focused aim and review question can result in selected studies with very similar focuses [39, 49], whereas a broad aim and question can result in heterogeneous studies with no clear commonalities making analysis and synthesis challenging [49, 53]. For example, Erasmus [50] selected four studies with very diverse topics, aims and research populations which led to difficulties synthesising them. Highly dissimilar studies might not be suited to synthesis using meta-ethnography at all [49, 53]; there is little reason to synthesise studies with no common ground. A meta-ethnography requires commensurate studies with compatible aims [39, 49]. However, to develop a full understanding of the phenomenon reviewers must also consider refutational data [39, 54, 55], thus a balance between homogeneity and heterogeneity of studies is needed [53]. Having a large volume of accounts to synthesise has arisen mainly due to reviewers adopting systematic review comprehensive literature search and selection methods.

### Phase 5. “Translating the studies Into One Another”

Data from 41 publications were coded at the node Phase 5 with 12 of them – 10 from health disciplines - providing rich detail (see Additional file 2: Table S2). The goal of Phase 5 has been described as the attempt to translate concepts from one study into another in order to arrive at concepts or metaphors which embody more than one study [49, 56]. Noblit and Hare [9], and others subsequently [1, 39, 46], have described the process of translation as fundamental to conducting a meta-ethnography; it appears to be unique to meta-ethnography compared to other qualitative synthesis methodologies [1]. There are two types of translation described by Noblit and Hare [9] for synthesising data in a meta-ethnography: reciprocal translation and refutational translation. The third method of synthesis, line of argument, is described by us under phase 6 because we see it as part of the interpretive synthesis process which comes after phase 5. More has been written about reciprocal than refutational translation in the publications in our review, probably because the former is more commonly conducted and published [10, 20, 38, 54, 57, 58]. We start by examining reciprocal translation.

### Reciprocal translation

The publications contributing the most material to this section were some of the worked examples, six health-related and one from education [1, 19, 21, 37, 39, 50, 59]. Noblit and Hare [9] did not provide a step-by-step guide in how to conduct translation [21], leaving some processes open to interpretation (and innovation) in how they were operationalised [1, 60]. Our analysis identified several different ways of conducting reciprocal translation and three possible types of process we have labelled A, B and C (not all of which appear in every meta-ethnography and processes A and B are not necessarily sequential):

(A) organise (group and/or order) the study accounts,
(B) organise (e.g. group) the concepts, themes, or metaphors from accounts,
(C) and analyse (translate) the conceptual data.

We now describe each type of process (A, B and C) in turn.

A. Organise the study accounts
  Some authors first grouped study accounts, such as by topic focus [1, 38, 61], before ordering them, for example, chronologically, within those groups to deal with a large volume of heterogenous data. In contrast, others, such as Atkins et al. [21], started translation with the earliest published ‘index’ study [1, 21, 45, 49, 61], and then ordered accounts chronologically. Others chose a ‘conceptually rich’ index account [60, 61]. In either strategy (starting with the richest or earliest study), concepts from each account were compared and contrasted against the index account. Different ways of ordering study accounts for translation have not been formally empirically compared [10], and there is no guidance for reviewers. It is not clear how a ‘rich’ index account should be selected [19]. The order could profoundly affect the synthesis output: concepts from one study could affect interpretation of concepts from other studies and thus the overall interpretation [10, 19, 21, 34, 49, 60]. Toye et al. [19] did not order accounts for translation seeing it as unmanageable for 77 accounts.
B. Organise the concepts, themes, metaphors
  Another possible process in Phase 5 is organising primary study authors’ conceptual data (‘second order constructs’) thematically [19, 21, 39, 50], e.g. by grouping concepts with similar meanings [19, 21, 39].
C. Translation of data
  The next step is to start translation. Authors of several publications in our review, similar to Noblit and Hare [9], likened translation to the constant comparative method used in grounded theory [1, 19, 38, 46, 49, 56, 59]. One approach to translation is to compare concepts individually account by account [1, 38, 46, 49, 56, 59, 61], for instance, the research group including Campbell, Britten, Pope and colleagues [1, 38, 46, 49, 56] outlined a systematic method, close to how Noblit and Hare [9] described it for synthesising ethnographies. When synthesising published journal articles, they compared the concepts in account 1 to those of account 2, the synthesis of those 2 accounts with account 3, and so on. Atkins et al. [21] followed a similar process, although they compared account by account within the categories they had developed from their thematic analysis. Alternatively, Doyle [59] operationalised translation as the writing of a ‘descriptive narrative’ ([59], p. 332) for each of four ethnographic case studies. Her narratives were based on her identification of hundreds of metaphors, defined as ‘salient language’ ([59], p. 333), they each contained.

In contrast, Toye et al. [19] chose not to compare concepts account by account because they had a large volume of data to synthesise. They sorted concepts into conceptual categories which they discussed and further interpreted as a team, i.e. they grouped and compared concepts. This method appears to diverge most from that of Noblit and Hare [9]. Comparing concepts has also been used for updating an existing meta-ethnography [62] by adapting Noblit and Hare’s methods which were designed for conducting a one-off meta-ethnography.

#### Insights on reciprocal translation

There is more than one way to conduct reciprocal translation. Reviewers have interpreted and/or adapted the methods to suit their particular purposes and data. A criticism regarding the conduct of reciprocal translation is that it can be done in such a way as to result in a simple

‘re-coding and re-categorizing qualitative findings and identifying alternative categorizations’ ([58], p. 1586)

from the primary studies rather than being interpretive [58]. Reciprocal translation may be interpretive to a greater or lesser extent, depending on the process used, and may end up, as Noblit puts it

‘producing reciprocal syntheses that are the product of the “dominant set of ideas” logic of social science’ (Noblit in Thorne et al. [20], p. 1348).

This could be a risk with approaches which focus predominantly on identifying commonalities, such as through grouping common concepts; the trend in health sciences for synthesising large numbers of journal articles has undoubtedly contributed to the adoption of such approaches.

### Refutational translation

Since Noblit and Hare’s book [9], which described examples of ethnographic studies with refutational ideologies, some authors have proposed that refutation may involve comparing contradictory themes, concepts or findings within or across study accounts (e.g. [58]), not just the overall conclusions or underlying ideologies of the accounts [1]. It is likely that all these types of refutation exist. Moreover, Campbell et al. [1] found that one meta-ethnography can include reciprocal and refutational (and line of argument) syntheses, not just one type as Noblit and Hare [9] implied. Some apparently reciprocal translations contain elements of refutation [62].

According to authors of our review publications, the purpose of refutational translation is to explore and explain differences, contradictions and exceptions in the studies [1, 19, 38, 46, 49–51, 53, 54, 56–58, 60, 63]. Meta-ethnography is described as one of the few qualitative evidence synthesis methods which requires the researcher

‘to give explicit attention to identification of incongruities and inconsistencies’ [54], p. 128).

These ‘deviant data’ are important because they can potentially lead to new understandings [51].

Finfgeld-Connett [58] suggested that refutational translation may be operationalised by placing two refutational concepts at either end of a continuum and then analysing differences among the concepts. She identified an additional approach for expressing refutational findings: create a narrative or ‘storyline’ so that ‘findings are placed into context’ ([58], p. 1589).

#### Insights on refutational translation

Published examples of refutational translation appear to be rare [10, 20]; reviewers often focus on shared themes/ findings within study accounts [20, 54]. Another issue is that reviewers may not label a refutational synthesis as such (e.g. [60]). Among our review publications, we saw two examples of refutational translation [38, 60]. Garside [60] conducted a meta-ethnography on women’s experiences of heavy menstrual bleeding (HMB), which she herself did not describe as refutational. Nonetheless, she identified refutational findings and found a disjoint between a biomedical and a lay model of HMB. This meta-ethnography was identified as refutational by members of our stakeholder group. Another example is a meta-ethnography in which the reviewers had difficulty reciprocally translating an account which had used a biomedical theoretical framework to analyse data deductively [38]. We propose that deductive primary qualitative research is not suited to synthesis using an inductive, interpretive methodology such as meta-ethnography; although a priori theories can still be used to inform the analytic synthesis.

### Phase 6. Synthesising translations

Data from 33 publications, shown in Additional file 2: Table S2, were coded at the node Phase 6. Phase 6 was described in the review publications as aiming to provide a fresh interpretation of phenomena through developing new findings or a new conceptualisation (e.g. [21, 39, 44, 50, 59]). In contrast to how Noblit and Hare [9] described Phase 6, we consider that it has two aspects: synthesising translations *and* line of argument synthesis. Noblit and Hare [9] initially said that you *either* do a line of argument *or* a reciprocal or refutational synthesis, but they also said that you conduct translation *before* doing a line of argument synthesis*.* This has undoubtedly led to confusion among researchers. It is possible that the term ‘line of argument’ was used by Noblit and Hare to describe two similar but different processes and synthesis products (one following from phase 4 and one following from phase 5). Their description of line of argument synthesis was:

‘What can we say of the whole (organization, culture, etc.), based on selective studies of the parts? This is the same as basic theorizing in qualitative research and is conceptualized alternatively as clinical inference and grounded theorizing.’ ([9], p. 63).

Here Noblit and Hare drew a parallel between developing a line of argument and developing a grounded theory [64]. In 2004, Noblit [20] described line of argument synthesis as constructing an argument about what a set of studies say. In meetings with the eMERGe project team and in a recent public lecture which the team organised, Noblit [65] clarified that a line of argument is a new ‘storyline’ or overarching explanation of a phenomenon. We propose that line of argument synthesis belongs in the later stages of meta-ethnography conduct and consequently have placed it in phase 6, subsequent to the processes of translating studies into one another and synthesising translations.

Definitions and understandings of line of argument synthesis are diverse, which was reflected in our multi-disciplinary stakeholder discussions. It has been described as a picture of the whole based on studies of the parts [1, 9, 19, 38, 44, 49, 50, 53, 56, 57, 66] and as being about inference [1, 9, 44, 49, 56, 66]. It has also been described as: a new or ‘higher order’ interpretation (like hypothesis generation) [21, 38, 67, 68], a mid-range theory [61], the development of a new overarching model [21], and/or a form of grounded theory [1, 9]. We maintain that these definitions are not necessarily incompatible with one another, for example, inference could lead to a new interpretation, explanation, model, theory or hypothesis; and new interpretations, explanations, models, theories and hypotheses are all potential outputs of a meta-ethnography. In addition, different terms (model, theory etc.) may be given to the same output depending on the author’s academic discipline or personal preference.

Eight worked examples, all but one health-related, gave varying levels of details of conducting a synthesis of translations and/or a line of argument synthesis [1, 19, 21, 37–39, 50, 59, 62]. The process used for synthesising translations varied and depended on how the studies related to one another (Phase 4) and on the way phase 5 was conducted, however, there are some broad commonalities. A process of reading and interpreting the phase 5 translations in order to produce a textual synthesis or narrative/storyline which expressed a new conceptualisation [1, 19, 21, 37, 39, 59], was often combined with (and preceded by) visual diagrams and models showing concepts and their inter-relationships [1, 19, 21, 37]. Where study accounts had been grouped and translated within those groups, phase 6 involved pulling together findings from across all the groups and accounts (e.g. [1, 37]). We observed that there may be multiple lines of argument resulting from a meta-ethnography, reflecting the complexity of qualitative research findings [1, 39, 60]. These lines of argument could be combined into one theory. Detailed examples of conduct of phase 6 are given for four meta-ethnographies [1, 19, 21, 37] in the illustrative case studies.

Various formats for the synthesis processes and outputs (findings) of phase 6 were seen in review publications. The worked examples (e.g. [1, 37]) usually used multiple formats including: visual, e.g. figures, graphics, diagrams [19, 37, 62]; models [1, 19, 21, 37, 60]; a textual line of argument [1, 21, 38, 39, 59]; hypotheses [21]; third order concepts/ synthesised concepts [39, 62]; and middle-range theory [39].

#### Insights on phase 6

Phase 6 is not always carried out or reported in published meta-ethnographies [10] yet a key strength of meta-ethnography is that it can be used to produce a new interpretation (or configuration) of data, for instance, a conceptual understanding, theory, and/or model [1, 19, 21, 37, 55, 69]. We consider that phase 6 is a key part of meta-ethnography which should strive to move beyond developing new themes or concepts to theory development.

There are different disciplinary understandings of the term ‘theory.’ Britten et al. [39] claimed that their worked example produced middle-range theories in the form of hypotheses that could be tested by other researchers and in Britten and Pope [38] they also drew a parallel between a ‘lines-of-argument’ synthesis and hypothesis generation. However, Finfgeld-Connett [58] sees a line of argument as distinct from theoretical models with the latter being more comprehensive. Models might also be used to achieve theory development [47]. We regard theory as an explanatory framework which can account for all the synthesis findings. Hammersley [63] disputed that meta-ethnography can lead to new theories because further primary data cannot be collected. However, we contend that purposive sampling of study accounts could provide this kind of further data collection, provided there are suitable qualitative studies available and the reviewers have relevant expertise of the topic and the methodology. Consultation with relevant stakeholder groups could also be used to support theory development.

### Illustrative case studies of conduct of phases 4 to 6

We selected four worked examples of meta-ethnographies [1, 19, 21, 37] from those included in our systematic review as illustrative case study examples of how phases 4 to 6 have been conducted. The worked examples were selected on the basis of their rich descriptions of methods and use of contrasting approaches to the complex phases 4 to 6. Additional file 4: Table S4 summarises the four approaches.

#### Case study 1: Campbell et al. 2011

In 2011 Campbell et al. [1] published a lengthy methodological report for the UK National Institute of Health Research evaluating meta-ethnography as a method of qualitative synthesis for health care. They conducted meta-ethnographies in two contexts: (a) living with rheumatoid arthritis and (b) lay beliefs about medicine-taking in chronic disease. The report drew together their work, some of which had been published earlier [5, 38, 39, 49], including one meta-ethnography [5] which did not meet inclusion criteria for our review. Next we describe their methods for phases 4 to 6.

### Phase 4

#### Listing and juxtaposing concepts, themes, metaphors

Campbell et al. [1, 49] created hand-written lists of summarised concepts and findings from study accounts (published journal articles) and then drew lines and arrows between related concepts in the various studies.

#### Relating studies

They [1, 49] then used their lists of concepts and connecting arrows to identify groupings or ‘sets’ of studies with a common focus and aim within the broader topic of each of their meta-ethnographies. For instance, in a meta-ethnography on experiences of having diabetes [49], one group of accounts explored the ways in which people responded to diabetes and treatment regimens and the other investigated differences between patients’ and practitioners’ models of diabetes i.e., the groups of studies were looking at different aspects of the topic of interest. For another meta-ethnography on medicine taking, they grouped accounts by type of medicine, e.g. asthma medicines, anti-hypertensives, and then ordered accounts chronologically within medicine groups.

### Phase 5

#### Conducting translation comparing concepts account by account

Campbell et al. [1] outlined a step-by-step, systematic translation method, close to how Noblit and Hare [9] described it for ethnographies. Within each group of papers they proceeded as follows:

“paper 1 […] might have findings X, Y and Z. Paper 2 […] might have finding w (something new that was not in paper 1), findings x and y (findings similar to findings X and Y in paper 1) and nothing like finding Z from paper 1. So this would produce a synthesis of papers 1 and 2:

- finding w (from paper 2)
- findings X and x (from papers 1 and 2)
- findings Y and y (from papers 1 and 2)
- finding Z (from paper 1).

This synthesis of papers 1 and 2 would then be compared with paper 3 in the same way. Then the synthesis of papers 1, 2 and 3 would be compared with paper 4, and so on until all the studies […] had been translated into each other.” ([1], p. 57).

Campbell et al. [1] offered a detailed explanation of how they related the studies; this involved two steps: determining how findings related to each other within groups of studies, and determining how studies were related across groups. The team first created visual ‘maps’ or diagrams to summarise key findings onto a single page for each group and drew the relationships between findings. The maps also showed how the findings/concepts translated into one another and links between findings.

### Phase 6

They then compared the maps across the groups of studies in order to develop a model to encompass and give an overview of all the findings from all studies. Next, they synthesised the detailed textual translations across all the medicine groups: they repeatedly read the syntheses for each of the medicine groups then analysed the data thematically in

‘a process of interpretation and conceptual advancement’ ([1], p. 64).

They described the process as

‘a continuous comparative analysis of texts until a comprehensive understanding of the phenomenon is reached’ ([1], p. 11).

They produced an overaching ‘textual synthesis’ forming a new conceptualisation. They called this a line of argument synthesis, which they equated with a third order interpretation.

In summary, the steps they took were:

1. Organise studies into medicine groups,
2. translate studies within medicine groups resulting in a textual synthesis for each group (reciprocal translations)
3. determine how the findings relate within medicine groups to produce medicine maps and across medicine groups to produce an overall model of medicine taking
4. synthesising translations across medicine groups to produce an overall textual synthesis of medicine taking.

Additional file 4: Table S4 shows a summary of their approach.

#### Case study 2: Atkins et al. 2008

Atkins et al. [21] published a worked example of their meta-ethnography, which synthesised 44 study accounts (journal articles), conducted to determine barriers to and facilitators of tuberculosis (TB) treatment adherence.

### Phase 4

They had a relatively large number of accounts containing disparate concepts. They therefore decided to conduct a ‘thematic analysis’ (after Pound et al. [5]), gathering similar themes into categories, to reveal commonalities prior to translating themes within those categories in Phase 5.

### Phase 5

They then arranged accounts chronologically in order to take into account any impact of policy changes on TB disease management, although ultimately the timing of policy changes was poorly reported in the study accounts. They compared the themes in account 1 to those of account 2, the synthesis of those 2 accounts with account 3, and so on, within the categories they had developed from their thematic analysis

“thematic analysis of themes identified in step 3 [was used] to identify nine categories, closely mimicking Pound et al. [14]. These categories included, for example, “social factors”, “disease progress” and “financial burden”, and the data within each category formed the basis for the reciprocal translation”. ([21], p. 6).

Similar to Campbell et al’s [1] method, they compared concepts account by account and grouped accounts but, in contrast to Campbell et al. [1], Atkins et al. did this by the thematic categories they had developed rather than by study topic.

### Phase 6

Atkins et al. [21] moved from translation to developing models in order to form a line of argument. They reinterpreted the meaning of studies and formulated hypotheses.

‘In developing an overarching model (or third order interpretation or synthesis), we listed the translated themes and subthemes in a table, juxtaposed with secondary themes derived from author interpretations. Each member of the (multi-disciplinary) research team then independently developed an overarching model that linked together the translations and authors’ interpretations. These models were then merged, discussed, and used to generate hypotheses, in order to produce a ‘line- of-argument’ synthesis. Each author was also asked to develop a mind map of their own model of the synthesis.’ ([21], pp. 7–8).

They produced hypotheses and a model of adherence to TB treatment.

Additional file 4: Table S4 shows a summary of their approach.

#### Case study 3: Malpass et al. 2009

The aim of Malpass et al’s [37] meta-ethnography was to derive new conceptual understandings of patients’ experiences of antidepressants. Their description of conducting phase 6 was particularly detailed.

### Phase 4

#### Relating studies

They [37] grouped study accounts (journal articles) by their focus, similar to Campbell et al. [1, 49] but in contrast to Atkins et al. [21]. Malpass et al. identified two groupings with different conceptual focuses relating to patients’ experience of antidepressants. They synthesised each of their groups separately before synthesising across the groups.

### Phase 5

They identified 33 common concepts from 16 accounts, for example, one of these was labelled ‘distressed and needing help.’ They then created a ‘summary definition’ ([37], p. 159), which is what they called translation, for each common concept e.g.

‘Recognition that something is seriously wrong, that self-help is not working and the experience of distress is beyond rational explanation.’ ([37], p. 159).

It is not clear if they compared concepts account by account (the method used by Campbell et al. [1]), nor how they arrived at the 33 common concepts.

### Phase 6

The authors [37] synthesised the translations for each of their two groups of accounts separately before pulling together those two separate syntheses into a final line of argument synthesis to construct ‘an overarching argument’ ([37], p. 161). This method was also used by Campbell et al. [1]. Malpass et al. [37] used a combination of visual graphics along with detailed textual description to convey their synthesised translations. For one group, they provided a complex flow chart displaying how patients are involved in a decision-making process linked to their evaluation of their anti-depressant use. For the second group, they provided a simpler visual diagram to show the impact of anti-depressant use on a person’s self-identity. Again detailed text described all aspects of the diagram. They also used a combination of visual graphics along with detailed textual description to convey their line of argument synthesis which involved bringing together the syntheses of the two groups of accounts. They displayed their line of argument visually by combining their two diagrams into a third diagram to convey the patients’ overall experience of taking anti-depressants. In addition they created a table to further explain two coping strategies related to managing anti-depressants from the line of argument diagram.

Additional file 4: Table S4 shows a summary of their approach.

#### Case study 4: Toye et al. 2014

Our final case study is Toye et al. [19] who produced a worked example to build on the methods of meta-ethnography and explore the challenges of synthesising a large number of qualitative studies. Their descriptions of Phases 4, 5 and 6 overlapped to a large degree because of their streamlined methods and processes.

### Phase 4

#### Listing and juxtaposing concepts, themes, metaphors

Unlike Campbell et al. [1], Atkins et al. [21] and Malpass et al. [37], Toye et al. [19] used qualitative analysis software NVivo 9 to record concepts. This involved three reviewers independently coding 450 conceptual findings from anywhere in 77 study accounts (journal articles). They coded using a hierarchical structure where the top-level ‘node’ or code was the study name and each sub-node was a concept from that study account; this enabled them to track from which study each concept came. Using NVivo’s functionality they also compared each reviewer’s node and coding structures.

Each of the three reviewers independently interpreted each concept and recorded this in a NVivo memo before comparing and combining these into one joint interpretation, which encompassed the study authors’ and their own intepretations. The joint interpretation was used as the conceptual data for phase 5, which was an innovative and unique approach. However, this approach could be criticised for moving away from the original authors’ terminology and thus potentially becoming less grounded in the original study’s context and meaning [1, 47, 59].

In contrast to the preceding three case studies [1, 21, 37], Toye et al. did not address how studies were related in Phase 4, because they moved immediately to analysing concepts; this could pose problems by resulting in trying to synthesise studies which are too dissimilar.

### Phase 5

Contrasting with Campbell et al’s approach to reciprocal translation [1], Toye et al. [19] consciously chose not to compare concepts account by account because it was unfeasible for the large number of studies, and hence concepts, they had to synthesise. They proceeded directly to organising concepts by sorting them into conceptual categories according to common meaning. For each category each team member wrote a description and a label (e.g. ‘body and self in conflict’). They discussed and further interpreted these conceptual categories as a team. They constantly compared the concepts looking for similarities and differences to ‘organise them into further abstracted conceptual categories’ ([19], p. 12).

### Phase 6

To conduct a line of argument synthesis, as a team they

‘collaboratively developed a visual structure of categories that made sense of the developing analysis’ ([19], p. 15)

by referring back to team discussions, the study accounts and their coding and analysis recorded in NVivo. They constructed and revised what they described as both a diagram and a model to develop and refine the line of argument until the diagram/model expressed their joint interpretation.

Using NVivo is highly efficient, does not constrain analysis through use of a starting ‘index’ study, and provides an audit trail to a large extent. However, potentially it is harder to keep track of the relationships between concepts within each study because of the number of concepts and the architecture of NVivo: once the move is made to recording interpretations in memos attached to specific sub-nodes (under which each study concept is coded), you lose track of which study that memo is linked to. Also the context of each study is harder to keep in mind with a large number of studies, especially when translation is done at the level of grouping concepts disembodied from their source accounts. Nonetheless, Toye et al. [19] claim that their collaborative interpretation of concepts helped them to be grounded in the studies because it

“challenge[d] our individual interpretations.” ([19], p. 8).

Additional file 4: Table S4 summarises the approach.

#### Summative discussion of case studies

Campbell et al’s [1, 49, 56] process is labour-intensive and is perhaps suited to a smaller volume of data in terms of number of accounts and/or amount of conceptual data but it facilitates immersion in the primary studies’ contexts and data. Toye et al’s [19] approach is efficient and suited to a large volume of data relative to the research team size [24]. However translating by grouping concepts at an early stage of analysis and synthesis with no account by account comparison might risk losing sight of the study foci, contexts and original conceptual meanings. Conducting a preliminary thematic categorisation of concepts, as Atkins et al. [21] did, might constrain subsequent translation and synthesis but can enable synthesis of heterogenous data, e.g. studies with few common concepts.

## Discussion

This systematic review provides an in-depth analysis and critique of methodological publications on meta-ethnography conduct since 1988 when Noblit and Hare [9] published their seminal meta-ethnography monograph. It provides guidance on the conduct of meta-ethnography Phases 4 to 6 which involve relating, translating, and synthesising studies. We undertook comprehensive and expansive literature searches. We conducted a rigorous analysis of 57 publications involving a multi-disciplinary team including social scientists, academic health professionals, lay people and other users of research evidence.

Our findings indicate that there is no ‘one size fits all’ recipe for reviewers to follow when conducting reciprocal translation. Each research team conducting a meta-ethnography will need to select methods which suit: the review aim; the nature, e.g. heterogeneity, and volume of the data to be synthesised; and their resources, such as team size and expertise and the time available [23, 24]. Large amounts of data have been synthesised by grouping studies into smaller sets then synthesising within and then across the groups of studies [1, 37] or by using analysis software to manage analysis [19]. Alternatively reviewers could manage the volume and nature of the data by, for instance, purposefully sampling studies [70] to reduce the volume and to ensure studies are similar enough to synthesise while still providing opportunities for inclusion of refutational data.

A key consideration in meta-ethnography conduct is which studies to include. The nature of the primary study data available to synthesise is an important factor. Incorporating predominantly superficial or ‘thin’ descriptive data in a meta-ethnography is potentially problematic: further interpreting data which lack depth and detail is difficult. We define conceptual data as explanatory, i.e. they explain a phenomenon. Rich descriptive data are those which provide sufficient detail that they can be further interpreted to develop conceptual insights. Rather than including ever-increasing volumes of studies based on topic relevance alone, selecting studies containing data suitable for a meta-ethnography is potentially more conducive to producing an interpretive synthesis.

The process of translation we, and others [1], believe is what distinguishes meta-ethnography from other qualitative evidence synthesis methodologies, therefore we propose it should be done using the theoretical principles laid out by Noblit and Hare [9]. Less labour-intensive methods of translation, such as grouping concepts without an account by account comparison (e.g. used by Toye et al. [19]), diverge more from Noblit and Hare’s original methodology. Nonetheless, such methods are likely to be popular with reviewers in light of the trend in health sciences towards synthesising high numbers of journal articles into a single meta-ethnography, e.g. over 100 in some published examples [71]. This is not a development we would advocate because the sheer volume of data might interfere with the ability to produce a useful, interpretive output and could result in an aggregative synthesis. There is a need to empirically compare alternative methods of synthesis to deal with large amounts of data. The order in which studies are synthesised could also influence the overall interpretation [10, 19, 21, 34, 49, 60], this too requires empirical investigation. Reviewers choosing methods for phases 4 to 6 should consider their potential impact on not only the efficiency of conduct, but also the outputs of a meta-ethnography.

We maintain that different kinds of syntheses (reciprocal, refutational and line of argument) can, and should, co-exist in one meta-ethnography [1], rather than it containing only one of these. Indeed in his new book, which credits his discussions with the eMERGe team, Noblit [72] accedes that these are not mutually exclusive types of syntheses. Refutational data are important for developing new understandings. We believe that theory development is of key importance to meta-ethnography conduct and that capitalising on the ability of meta-ethnography to move beyond the development of new concepts to theory development should enhance the evidence base for decision making. The methodology is suited to complex data and complex questions. If reviewers do not intend to develop theory, then an alternative qualitative evidence synthesis methodology could be better suited to their purposes.

Since conducting our systematic review in 2015 to 2016 further relevant publications have been published. For instance, they include adapting meta-ethnography for synthesising qualitative evidence syntheses (‘mega-ethnography’) [73]; for analysing multiple primary qualitative datasets [74], and for synthesising ethnographies while they were still being conducted [75]. Urrieta and Noblit’s new edited book [72] focuses on the relation of meta-ethnography and theory with identity theory. It also explores how meta-ethnography has been adapted in health and in education and clarifies some ambiguities in Noblit and Hare’s 1988 book [9]. After our systematic review was completed Cahill et al. [76] produced a guide entitled ‘A guide to conducting a meta-ethnography’ in 2018. Their article gave a concise overview of meta-ethnography conduct based on only 10 publications, all included in our systematic review, but did not provide in-depth analysis or guidance on conducting phases 4 to 6, which is the main focus of our article.

Meta-ethnographies conducted in education versus health and social care disciplines may evolve distinct versions of the methodology to suit their different needs and philosophical approaches. A special issue in the journal *Ethnography and Education* in 2017 [77] reflects a new interest in meta-ethnography in the field of education; in this, Borgnakke [78] challenges the transferability of the ‘evidence movement’ basis of meta-ethnography in healthcare research to education and social fields. Education tends towards synthesis of a small number of rich ethnographies and the identification of metaphors (e.g. [59, 75]), whereas health science tends to synthesise concepts and themes from large numbers of journal articles reporting interview studies [10]. Meta-ethnography is still evolving, in health and other disciplines, and future research by our team will seek to incorporate these newer publications, not all of which can be covered here, into future guidance on meta-ethnography conduct and reporting.

We identified a lack of empirical methodological research comparing the different methods of relating studies, translation and synthesis meaning that there remain unanswered questions. Future methodological research should focus on establishing the consequences of different methods for the quality (e.g. credibility and trustworthiness) of meta-ethnography outputs, such as, the impact of grouping concepts thematically compared to translating them one by one. In addition, research should explore the impact of the order in which accounts are translated and synthesised, including the effect of using an index study. A further issue to examine is the relationship between volume of data and quality of output.

### Limitations

We originally conducted our systematic review to inform development of reporting guidance [22–27]. In order to discern what should be reported in a meta-ethnography, we had to establish how a meta-ethnography should be conducted and hence many publications contained rich data on meta-ethnography conduct. A possible limitation is the lack of formal methodological evidence, however we critiqued the methods through comparing and contrasting them and reflected through a process of logical reasoning. Not all publications in the review contributed to the findings, especially for phases 5 and 6; some, such as worked examples of meta-ethnographies containing methodological detail, did contribute rich data. However, other kinds of texts in our review, such as overviews of the methodology, also contributed to our understanding and analysis. Our review included publications up to 2016. We have since updated our systematic searches in five databases (CINAHL, Web of Science, PubMed, SCOPUS and PsychInfo) during June and July 2018 and also identified publications through citation alerts and our networks. Newer publications have been incorporated into the discussion section.

We chose not to critically appraise texts in order to exclude any on the basis of (low) quality. No tool exists to judge the quality of a meta-ethnography, nor the quality of such a wide range of methodological publications which ranged from worked examples to critiques and overviews of the methodology. We did however record which ones we considered to be rich in detail. A publication’s richness is reflected in how much it contributed to our review findings. It is worth mentioning that one worked example, which contributed to our findings, was conducted by a lone reviewer [50]; good practice is for multiple reviewers in order to enhance interpretation of data [24].

A further limitation is the lack of clarity around conduct of Phase 6 and line of argument synthesis in the review publications. This reflects its complexity and unclear guidance on its conduct. There was lots of variation in definitions and no clear consensus on methods, although we could discern some commonalities. There is scope for future research to further develop methods for conducting phase 6 and line of argument synthesis.

## Conclusions

Thinking and practice in meta-ethnography conduct has developed and continue to evolve as it is applied in new ways. There are various different methods for conducting the analytic synthesis in a meta-ethnography but empirical methodological research is required to evaluate them. Researchers conducting a meta-ethnography will need to select methods which suit their particular purpose and data, bearing in mind the potential impacts of those methods on the quality of output. Our work should assist those planning and conducting meta-ethnographies to design and carry out their synthesis. Ultimately better conducted (and reported) meta-ethnographies will better contribute to evidence-based practice.

## Additional files

Additional file 1: Databases and sources searched in June–August 2015 for systematic review. A list of the bibliographic databases and other online sources searched in the systematic review. (DOCX 19 kb)

Additional file 2: **Table S2.** Characteristics of review publications and their contributions to phases 4, 5 and 6. A table summarising the characteristics of the publications included in the systematic review including whether they provided material that was relevant to phases 4–6 of meta-ethnogrpahy conduct and if so whether it was rich in detail. (DOCX 103 kb)

Additional file 3: **Table S3.** Examples of ‘rich’ and ‘not rich’ data on phases 4 to 6 from systematic review publications. A table providing data excerpts from review publications illustrating data that we judged rich and not rich on conduct of phases 4 to 6 and explaining why. (DOCX 26 kb)

Additional file 4: **Table S4.** Comparative case studies of four meta-ethnography worked examples. A table comparing how phases 4 to 6 were conducted in four ‘worked example’ review publications. (DOCX 22 kb)

## Abbreviations

HMB: Heavy menstrual bleeding
NICE: National Institute for Health and Clinical Excellence
TB: Tuberculosis

## References

[1] Campbell R, Pound P, Morgan M, Daker-White G, Britten N, Pill R, Yardley L, Pope C, Donovan J (2011). Evaluating meta-ethnography: systematic analysis and synthesis of qualitative research. Health Technol Assess.

[2] Dalton J, Booth A, Noyes J, Sowden AJ (2017). Potential value of systematic reviews of qualitative evidence in informing user-centred health and social care: findings from a descriptive overview. J Clin Epidemiol..

[3] Lewin S, Booth A, Glenton C, Munthe-Kaas H, Rashidian A, Wainwright M, Bohren MA, Tunçalp Ö, Colvin CJ, Garside R (2018). Applying GRADE-CERQual to qualitative evidence synthesis findings: introduction to the series. Implement Sci.

[4] Nunes V, Neilson J, O’Flynn N, Calvert N, Kuntze S, Smithson H, Benson J, Blair J, Bowser A, Clyne W (2009). Clinical guidelines and evidence review for medicines adherence: involving patients in decisions about prescribed medicines and supporting adherence.

[5] Pound P, Britten N, Morgan M, Yardley L, Pope C, Daker-White G, Campbell R (2005). Resisting medicines: a synthesis of qualitative studies of medicine taking. Soc Sci Med.

[6] Downe S, Finlayson K, Tunçalp Ö, Gülmezoglu A. Factors that influence the uptake of routine antenatal services by pregnant women: a qualitative evidence synthesis. Cochrane Database of Systematic Reviews. 2016;10. Art. No.: CD012392. 10.1002/14651858.CD012392. 10.1002/14651858.CD012392.pub2 PMC6564082 31194903

[7] Cohen EE, LaMonte SJ, Erb NL, Beckman KL, Sadeghi N, Hutcheson KA, Stubblefield MD, Abbott DM, Fisher PS, Stein KD (2016). American Cancer Society head and neck cancer survivorship care guideline. CA Cancer J Clin.

[8] Lang H, France E, Williams B, Humphris G, Wells M (2013). The psychological experience of living with head and neck cancer: a systematic review and meta-synthesis. Psycho-Oncology.

[9] Noblit GW, Hare RD (1988). Meta-ethnography: synthesizing qualitative studies.

[10] France EF, Ring N, Thomas R, Noyes J, Maxwell M, Jepson R (2014). A methodological systematic review of what's wrong with meta-ethnography reporting. BMC Med Res Methodol.

[11] Dixon-Woods M, Booth A, Sutton AJ (2007). Synthesizing qualitative research: a review of published reports. Qual Res.

[12] Hannes K, Macaitis K (2012). A move to more systematic and transparent approaches in qualitative evidence synthesis: update on a review of published papers. Qual Res.

[13] Uny I, France EF, Noblit GW (2017). Steady and delayed: explaining the different development of meta-ethnography in health care and education. Ethnogr Educ.

[14] Geertz C (1973). The interpretation of cultures.

[15] Turner SP: Sociological Explanation As Translation; 1980.

[16] Noyes J, Booth A, Cargo M, Flemming K, Garside R, Hannes K, Harden A, Harris J, Lewin S, Pantoja T (2018). Cochrane qualitative and implementation methods group guidance series—paper 1: introduction. J Clin Epidemiol.

[17] SIGN, British Thoracic Society: SIGN 153 - British guideline on the management of asthma. A national clinical guideline. . In*.*; 2016.

[18] Ring NJ, R.;Hoskins, G.;Wilson, C.;Pinnock, H.;Sheikh, A.;Wyke, S.: Understanding what helps or hinders asthma action plan use: a systematic review and synthesis of the qualitative literature. Patient Educ Couns 2011, 85(2):e131–e143. 10.1016/j.pec.2011.01.025 21396793

[19] Toye F, Seers K, Allcock N, Briggs M, Carr E, Barker K (2014). Meta-ethnography 25 years on: challenges and insights for synthesising a large number of qualitative studies. BMC Med Res Methodol.

[20] Thorne S, Jensen L, Kearney MH, Noblit G, Sandelowski M (2004). Qualitative metasynthesis: reflections on methodological orientation and ideological agenda. Qual Health Res.

[21] Atkins S, Lewin S, Smith H, Engel M, Fretheim A, Volmink J (2008). Conducting a meta-ethnography of qualitative literature: lessons learnt. BMC Med Res Methodol.

[22] France EF, Ring N, Noyes J, Maxwell M, Jepson R, Duncan E, Turley R, Jones D, Uny I (2015). Protocol-developing meta-ethnography reporting guidelines (eMERGe). BMC Med Res Methodol.

[23] Cunningham M, France EF, Ring N, Uny I, Duncan EAS, Roberts RJ, Jepson RG, Maxwell M, Turley RL, Noyes J. 13/114/60. Developing a reporting guideline to improve meta-ethnography in health research: the eMERGe mixed-methods study. Health Services and Delivery Research Journal. In press. 30758932

[24] France EF, Cunningham M, Ring N, Uny I, Duncan EAS, Jepson RG, Maxwell M, Roberts RJ, Turley RL, Booth A, et al. Improving reporting of Meta-ethnography: the eMERGe reporting guidance. J Adv Nurs. Epublication ahead of Print. 15 January 2019. 10.1111/jan.13809. 10.1111/jan.13809 PMC7594209 30644123

[25] France EF, Cunningham M, Ring N, Uny I, Duncan EAS, Jepson RG, Maxwell M, Roberts RJ, Turley RL, Booth A, et al. Improving reporting of Meta-ethnography: the eMERGe reporting guidance. Review of Education. Epublication ahead of Print. 15 January 2019. 10.1002/rev3.3147.

[26] France EF, Cunningham M, Ring N, Uny I, Duncan EAS, Jepson RG, Maxwell M, Roberts RJ, Turley RL, Booth A, et al. Improving reporting of Meta-ethnography: the eMERGe reporting guidance. Psycho-oncology. Epublication ahead of Print. 15 January 2019. 10.1002/pon.4915. 10.1002/pon.4915 30644150

[27] France EF, Cunningham M, Ring N, Uny I, Duncan EAS, Jepson RG, Maxwell M, Roberts RJ, Turley RL, Booth A, et al. Improving reporting of Meta-Ethnography: The eMERGe Reporting Guidance. BMC Med Res Methodol. In press. 10.1186/s12874-018-0600-0 PMC6359764 30709371

[28] Finlayson KW, Dixon A (2008). Qualitative meta-synthesis: a guide for the novice. Nurse Res.

[29] Paterson BL. “It looks great but how do I know if it fits?”: an introduction to Meta-synthesis research. In K. Hannes & C. Lockwood (eds.): Synthesizing Qualitative Research. Chichester: Wiley; 2011. p. 1–20.

[30] Tong A, Flemming K, McInnes E, Oliver S, Craig J (2012). Enhancing transparency in reporting the synthesis of qualitative research: ENTREQ. BMC Med Res Methodol.

[31] Bearman M, Dawson P (2013). Qualitative synthesis and systematic review in health professions education. Med Educ.

[32] Toye F, Seers K, Allcock N, Briggs M, Carr E, Andrews J, Barker K (2013). Trying to pin down jelly' - exploring intuitive processes in quality assessment for meta-ethnography. BMC Med Res Methodol.

[33] Franzel B, Schwiegershausen M, Heusser P, Berger B (2013). How to locate and appraise qualitative research in complementary and alternative medicine. BMC Complement Altern Med.

[34] Booth A. Acknowledging a Dual Heritage for Qualitative Evidence Synthesis: Harnessing the Qualitative Research and Systematic Review Research Traditions. University of Sheffield; 2013. 10.1177/1049732318808247 30799763

[35] Carroll C, Booth A (2015). Quality assessment of qualitative evidence for systematic review and synthesis: is it meaningful, and if so, how should it be performed?. Res Synth Methods.

[36] Booth A, Noyes J, Flemming K, Gerhardus A, Wahlster P, Van Der Wilt G, Mozygemba K, Refolo P, Sacchini D, Tummers M, et al. Guidance on choosing qualitative evidence synthesis methods for use in health technology assessments of complex interventions. 2016. [Online]. Available from: http://www.integrate-hta.eu/downloads/.

[37] Malpass A, Shaw A, Sharp D, Walter F, Feder G, Ridd M, Kessler D (2009). **“**Medication career” or “moral career”? The two sides of managing antidepressants: a meta-ethnography of patients' experience of antidepressants. Soc Sci Med.

[38] Britten N, Pope C, Hannes K, Lockwood C (2012). Medicine taking for asthma: a worked example of meta-ethnography (chapter 3).

[39] Britten N, Campbell R, Pope C, Donovan J, Morgan M, Pill R (2002). Using meta ethnography to synthesise qualitative research: a worked example. J. Health Serv. Res. Policy.

[40] Suri H, Clarke D (2009). Advancements in research synthesis methods: from a methodologically inclusive perspective. Rev Educ Res.

[41] Sigurdson C, Woodgate R (2015). Designing a Metasynthesis study in pediatric oncology nursing research. J Pediatr Oncol Nurs.

[42] Beck CT (2009). Metasynthesis: a goldmine for evidence-based practice. AORN J.

[43] Bondas T, Hall EO (2007). Challenges in approaching metasynthesis research. Qual Health Res.

[44] Pope C, Mays N, Popay J. Chapter 4- interpretive approaches to evidence synthesis. In Pope C, Mays N, Popay J: Synthesizing qualitative and quantitative health evidence: a guide to methods. Maidenhead: Open University Press; 2007. p. 72–94.

[45] McCann S, Campbell M, Entwistle V (2013). Recruitment to clinical trials: a meta-ethnographic synthesis of studies of reasons for participation. J Health Serv Res Policy.

[46] Pope C, Mays N. Synthesising qualitative research. In: Pope C, Mays N, editors. Qualitative research in health care (3rd ed). Oxford : Blackwell Publishing; BMJ Books; 2006. p. 142–52.

[47] Bondas T, Hall EOC (2007). A decade of metasynthesis research in health sciences: a meta-method study. Int J Qual Stud Health Well Being.

[48] Melendez-Torres GJ, Grant S, Bonell C (2015). A systematic review and critical appraisal of qualitative metasynthetic practice in public health to develop a taxonomy of operations of reciprocal translation. Res Synth Methods.

[49] Campbell R, Pound P, Pope C, Britten N, Pill R, Morgan M, Donovan J (2003). Evaluating meta-ethnography: a synthesis of qualitative research on lay experiences of diabetes and diabetes care. Soc Sci Med.

[50] Erasmus E (2014). The use of street-level bureaucracy theory in health policy analysis in low- and middle-income countries: a meta-ethnographic synthesis. Health Policy Plan.

[51] Walsh D, Downe S (2005). Meta-synthesis method for qualitative research: a literature review. J Adv Nurs.

[52] McCormick J, Rodney P, Varcoe C (2003). Reinterpretations across studies: an approach to meta-analysis. Qual Health Res.

[53] Kinn LG, Holgersen H, Ekeland TJ, Davidson L (2013). Metasynthesis and bricolage: an artistic exercise of creating a collage of meaning. Qual Health Res.

[54] Booth A, Carroll C, Ilott I, Low LL, Cooper K (2013). Desperately seeking dissonance: identifying the disconfirming case in qualitative evidence synthesis. Qual Health Res.

[55] Meadows-Oliver M. Meta-ethnography. In: de Chesnay MdC M, editor. Nursing research using ethnography: Qualitative designs and methods in nursing. New York: Springer Publishing Co; 2015. p. 171–9.

[56] Campbell R, Britten N, Pound P, Donovan J, Morgan M, Pill R, Pope C. Section 4.8- using meta-ethnography to synthesise qualitative research. In: Popay J, editor. Moving beyond effectiveness in evidence synthesis: Methodological issues in the synthesis of diverse sources of evidence. London: NICE; 2006. p. 75–82.

[57] Barnett-Page E, Thomas J (2009). Methods for the synthesis of qualitative research: a critical review. BMC Med Res Methodol.

[58] Finfgeld-Connett D (2014). Metasynthesis findings: potential versus reality. Qual Health Res.

[59] Doyle LH (2003). Synthesis through meta-ethnography: paradoxes, enhancements, and possibilities. Qual Res.

[60] Garside R. A comparison of methods for the systematic review of qualitative research: two examples using Meta-ethnography and Meta-study. UK: University of Exeter; 2008.

[61] Nye E, Melendez-Torres GJ, Bonnell C (2016). Origins, methods, and advances in qualitative meta-synthesis. Review of Education.

[62] France EFW, Wells M, Lang H, Williams B (2016). Why, when and how to update a meta-ethnography qualitative synthesis. Systematic reviews.

[63] Hammersley M. Chapter 11- what is qualitative synthesis and why we do it? In M. Hammersley (ed.): The myth of research based policy and pratice. London: SAGE Publications Ltd; 2013; p. 130–151.

[64] Glaser B, Strauss A (1967). The discovery of grounded theory. London: Weidenfeld and Nicholson.

[65] Noblit GW: How qualitative (or interpretive or critical) is qualitative synthesis and what we can do about this? . In: A public lecture by Professor George W Noblit, University of North Carolina at Chapel Hill Edinburgh. Available at: http://emergeproject.org/wp-content/uploads/2016/09/How-qualitative.pdf; 2016.

[66] Hansen HP, Draborg E, Kristensen FB (2011). Exploring qualitative research synthesis: the role of patients' perspectives in health policy design and decision making. Patient.

[67] Dixon-Woods M, Agarwal S, Jones D, Young B, Sutton A (2005). Synthesising qualitative and quantitative evidence: a review of possible methods. J Health Serv Res Policy.

[68] Dixon-Woods M, Agarwal S, Young B, Jones D, Sutton AJ. Integrative approaches to qualitative and quantitative evidence. London: Health Development Agency; 2004.

[69] Noyes J, Lewin S, Noyes J, Booth A, Hannes K, Harden A, Harris J, Lewin S, Lockwood C, Cochrane collaboration qualitative methods Group (2011). Chapter 6: supplemental guidance on selecting a method of qualitative evidence synthesis, and integrating qualitative evidence with Cochrane intervention reviews. Supplementary Guidance for Inclusion of Qualitative Research in Cochrane Systematic Reviews of Interventions Version 1 (updated August 2011).

[70] Benoot C, Hannes K, Bilsen J (2016). The use of purposeful sampling in a qualitative evidence synthesis: a worked example on sexual adjustment to a cancer trajectory. BMC Med Res Methodol.

[71] Colvin CJ, Smith HJ, Swartz A, Ahs JW, de Heer J, Opiyo N, Kim JC, Marraccini T, George A (2013). Understanding careseeking for child illness in sub-Saharan Africa: a systematic review and conceptual framework based on qualitative research of household recognition and response to child diarrhoea, pneumonia and malaria. Soc Sci Med.

[72] Urrieta L Jr, Noblit GW. Cultural constructions of identity: Meta-ethnography and theory. New York: Oxford University Press; 2018.

[73] Toye F, Seers K, Hannink E, Barker K. A mega-ethnography of eleven qualitative evidence syntheses exploring the experience of living with chronic non-malignant pain. BMC Med Res Methodol. 2017;17(1):116. 10.1186/s12874-017-0392-7 PMC5540410 28764666

[74] Pilkington H (2018). Employing meta-ethnography in the analysis of qualitative data sets on youth activism: a new tool for transnational research projects?. Qual Res.

[75] Kakos M, Fritzsche B (2017). A meta-ethnography of two studies on interactions in schools: reflections on the process of translation. Ethnogr Educ.

[76] Cahill M, Robinson K, Pettigrew J, Galvin R, Stanley M. Qualitative synthesis: a guide to conducting a meta-ethnography. Br J Occup Ther. 2018;81(3):129-137.

[77] Kakos M, Fritzsche B (2017). Meta-ethnography E&E. Ethnogr Educ.

[78] Borgnakke K (2017). Meta-ethnography and systematic reviews–linked to the evidence movement and caught in a dilemma. Ethnogr Educ.

