# Supplementary material for: A methodological systematic review of meta-ethnography conduct to articulate the complex analytical phases
Source: BMC Med Res Methodol. 2019 Feb 18;19:35. doi: 10.1186/s12874-019-0670-7 (PMC6380066; doi:10.1186/s12874-019-0670-7)
Supplement: Supplementary file 1 — Databases and sources searched in June–August 2015 for systematic review. A list of the bibliographic databases and other online sources searched in the systematic review. (DOCX 19 kb) [file 12874_2019_670_MOESM1_ESM.docx]

Databases and resources searched in June-August 2015 for systematic review

| Bibliographic Databases:   - Applied Social Sciences Index and Abstracts (inception to 27/07/2015) - Australian Education Index (inception to 28/07/2015) - British Education Index (inception to 04/08/2015) - CINAHL (inception to 03/08/2015) - Educational Research Abstracts ERA (inception to 28/8/2015) - ERIC-Educational Resources Information Center) (inception to 27/07/2015) - EThOS (e-theses online service) (inception to 16/06/15) - International Bibliography of the Social Sciences (inception to 27/07/2015) - MEDLINE (1947 to 21/07/2015) - PsycINFO (inception to 03/08/2015) - Pubmed (inception to 03/08/15) - SCOPUS (1987 to 18/08/2015) - Sociological abstracts (inception to 27/07/2015) - Web of Science Core Collection (inception to 13/08/2015)   Online resources:   - Campbell Collaboration - Cochrane Collaboration - Open Grey - **CRD (Centre for Reviews and Dissemination)** - NIHR Journals Library |
| --- |
