# Supplementary material for: A methodological systematic review of meta-ethnography conduct to articulate the complex analytical phases
Source: BMC Med Res Methodol. 2019 Feb 18;19:35. doi: 10.1186/s12874-019-0670-7 (PMC6380066; doi:10.1186/s12874-019-0670-7)
Supplement: Supplementary file 2 — Table S2. Characteristics of review publications and their contributions to phases 4, 5 and 6. A table summarising the characteristics of the publications included in the systematic review including whether they provided material that was relevant to phases 4–6 of meta-ethnogrpahy conduct and if so whether it was rich in detail. (DOCX 103 kb) [file 12874_2019_670_MOESM2_ESM.docx]

Table 2. Characteristics of review publications and their contributions to phases 4, 5 and 6

|  |  |  |  |  | **Phase 4** | | **Phase 5** | | **Phase 6** | |
| --- | --- | --- | --- | --- | --- | --- | --- | --- | --- | --- |
| **Publication** | **Publication aim** | **Classification of data source** | **Discipline** | **Author(s) country of work** | **Contributes data?** | **Rich detail?** | **Contributes data?** | **Rich detail?** | **Contributes data?** | **Rich detail?** |
| Campbell *et al. 2011 (1)* | To appraise and synthesise qualitative health research for HTA using a meta-ethnographic approach | Methodological text, empirical  &  Meta-ethnographies with methodological detail | Health | UK | Yes | Yes | Yes | Yes | Yes | Yes |
| Hammersley 2013 (60) | How does qualitative synthesis differ from 'traditional' reviews? What does it add to primary research? What form of synthesis does it aim at? How can it contribute to the development of knowledge in a field? | Methodological text, opinion-based | Education | UK | No | - | No | - | No | - |
| Kinn *et al.2013 (53)* | To explore the systematic and creative research processes involved in meta-synthesising. | Methodological text, empirical and opinion-based | Health & social work | USA | No | - | Yes | No | Yes | No |
| Lee *et al.2015 (45)* | To examine how meta-ethnography is conducted in practice, drawing upon experience of undertaking three meta-ethnographies. | Methodological text, empirical (literature review) | Health | UK | Yes | No | Yes | No | Yes | Yes |
| Carroll and Booth (28) | To conduct a narrative review of recent literature that researches or  Discusses the issue of quality assessment for QES | Methodological text, empirical (literature review) | Health | UK | No | - | No | - | No | - |
| Finfgeld-Connett 2014 (34) | What types of meta-synthesis findings have been produced since Noblit and Hare’s (1988) methodological guidelines and what types of findings should be produced in the future? | Methodological text, empirical | Health | USA | No | - | Yes | Yes | No | - |
| Nye *et al.2016 (42)* | Discusses the key philosophical and methodological issues for qualitative meta-synthesis | Methodological text, empirical | Social policy | UK | No | - | Yes | Yes | Yes | No |
| Booth 2013 (27) | To examine the state of qualitative synthesis methods for literature searching , quality assessment, and exploring heterogeneity | Methodological text, empirical | Health | UK | No | - | No | - | No | - |
| Finfgeld-Connett and Johnson 2013 (35) | A report of literature search strategies for the purpose of conducting knowledge-building and theory-generating qualitative systematic reviews | Methodological text, empirical (literature review) | Health | USA | No | - | No | - | No | - |
| Noblit and Hare 1988 (12) | How qualitative researchers ought to think about interpretive explanation and synthesise multiple studies | Methodological text, empirical | Education | USA | Yes | Yes | Yes | Yes | Yes | Yes |
| Thorne *et al. 2004 (57)* | An examination of the tensions between comparison and integration, deconstruction and synthesis, and reporting and integration within meta-synthesis | Methodological text | Health | USA & Canada | No | - | Yes | No | Yes | No |
| Booth *et al. 2013 (43)* | To provide an overview of how the disconfirming case has been handled in the meta-synthesis literature | Methodological text, empirical | Health | UK | No | - | Yes | Yes | Yes | No |
| Britten *et al. 2002 (14)* | To demonstrate the benefits of applying meta ethnography to the synthesis of qualitative research | Meta-ethnography with methodological detail (ME) | Health | UK | Yes | Yes | Yes | Yes | Yes | Yes |
| Campbell *et al. 2003 (32)* | To examine the feasibility of synthesising qualitative research | ME | Health | UK | Yes | Yes | Yes | No | Yes | No |
| Doyle 2003 (59) | To show how meta-ethnography is a dynamic methodology for the synthesis of qualitative research | ME | Education | USA | Yes | No | Yes | Yes | Yes | No |
| McCormick *et al.2003 (52)* | Describe the methods they used to create a qualitative meta- analysis | ME | Health | Canada | Yes | No | Yes | No | Yes | No |
| Campbell *et al.2006 (55)* | Illustrate meta-ethnography with two worked examples | ME | Health | UK | No | - | No | - | No | - |
| Atkins *et al.2008 (18)* | To determine barriers and facilitators of tuberculosis treatment adherence | ME | Health | South Africa | Yes | No | Yes | Yes | Yes | Yes |
| Garside 2008 (33) | To review, compare, develop and assess meta-ethnography and meta-study | ME | Health | UK | Yes | Yes | Yes | Yes | No | No |
| Malpass *et al.2009 (38)* | To derive new conceptual understandings of patients’ experiences of antidepressants. | ME | Health | UK | Yes | Yes | Yes | Yes | Yes | Yes |
| Britten and Pope 2012 (47) | To illustrate meta-ethnography by means of a worked example | ME | Health | UK | Yes | Yes | Yes | Yes | Yes | Yes |
| McCann *et al.2013 (51)* | Discusses the substantive findings and the methodological implications for updating meta- ethnographies | ME | Health | UK | Yes | No | Yes | No | No | - |
| Erasmus2014 (48) | How street-level bureaucracy theory has been used in the literature and provide an example of the application of meta-ethnography | ME | Health (policy) | South Africa | Yes | Yes | Yes | Yes | Yes | No |
| Toye *et al.2014 (20)* | To build on the methods of meta-ethnography and explore the challenges of including a large number of qualitative studies | ME | Health | UK & Canada | Yes | Yes | Yes | Yes | Yes | Yes |
| France *et al.2016 (39)* | To describe and critique methods for updating a meta-ethnography | ME | Health | UK | Yes | No | Yes | Yes | Yes | Yes |
| Toye *et al. 2013 (25)* | Explores quality appraisal within a meta-ethnography | ME | Health | UK & Canada | No | - | No | - | No | - |
| Franzel *et al.2013 (26)* | To present a case study of how to locate and appraise qualitative studies for the conduct of a meta-ethnography | ME | Health | Germany | No | - | No | - | No | - |
| Noyes and Lewin 2011 (15) | Guidance for authors wishing to synthesise qualitative evidence to inform, enhance or extend a Cochrane intervention review | Guidance on conduct, empirical | Health | UK & Norway | No | - | Yes | N o | No | - |
| Paterson 2011 (22) | A brief overview of the uses and evolution of qualitative evidence synthesis methods, including how the various synthesis methods compare to one another | Guidance on conduct, empirical | Health | Canada | No | - | No | - | No | - |
| Booth *et al.2016 (29)* | Summarises current thinking and practice in the choice of qualitative evidence synthesis methods for health technology assessments and systematic reviews | Guidance on conduct, empirical | Health | International | No | - | No | - | No | - |
| Pope and Mays 2006 (13) | Considers three of the main methods that can be used to synthesise qualitative research: narrative synthesis, cross-case analysis, and meta-ethnography | Descriptive overview | Health | UK | No | - | Yes | No | Yes | No |
| Pope and Popay 2007 (50) | Explores interpretive approaches to synthesis of qualitative data | Descriptive overview | Health | UK | Yes | No | Yes | Yes | Yes | No |
| Barnett-Page and Thomas 2009 (56) | To draw together and review the full range of methods of synthesis | Descriptive overview, compares QES | Education | UK | No | - | Yes | No | Yes | No |
| Beck 2009 (76) | Addresses meta-synthesis and its application to perioperative evidence-based care | Descriptive overview | Health | USA | Yes | No | Yes | No | Yes | No |
| Ring *et al.2010 (77)* | A guide to the methods used to synthesise qualitative research | Descriptive overview | Health | UK | No | - | No | - | No | - |
| Hansen *et al. 2011 (62)* | Focuses on qualitative research synthesis in eliciting patients’ perspective | Descriptive overview | Health | Denmark | No | - | Yes | No | No | - |
| Saini and Shlonsky 2012 (78) | To provide current standards, philosophical debates, and methods for understanding and conducting systematic qualitative syntheses | Descriptive overview | Social work | Canada & Australia | No | - | No | - | No | - |
| Meadows-Oliver 2015 (36) | Describe process and challenges of conducting a meta-synthesis | Descriptive overview | Health | USA | Yes | No | Yes | No | Yes | No |
| Seers 2015 (79) | To outline what a qualitative systematic review is and explore what it can contribute to our understanding of pain | Descriptive overview | Health | UK | No | - | Yes | No | No | - |
| Sigurdson and Woodgate 2015 (80) | To give an overview of meta-synthesis research for nurse researchers | Descriptive overview (literature review), compares QES | Health | Canada | Yes | No | Yes | No | No | - |
| Finlayson and Dixon 2008 (21) | An overview of qualitative meta-synthesis methods and explores some philosophical, methodological and terminological issues | Descriptive overview, compares QES | Health | UK | No | - | No | - | No | - |
| Bearman and Dawson 2013 (24) | An overview of qualitative synthesis in health professional education | Descriptive overview, compares QES | Health | Australia | No | - | No | - | No | - |
| Ring et al. 2011(81) | Presents the methods for synthesising qualitative research most used in health research | Descriptive overview, compares QES | Health | UK | No | - | No | - | No | - |
| Kangasniemi *et al.2012 (31)* | To increase the understanding of synthesis as a method | Descriptive overview | Health | Finland | Yes | No | Yes | No | Yes | No |
| Bondas and Hall 2007 (46) | To discuss meta-synthesis with the goal of understanding this research approach more fully | Critique, opinion-based; compares QES | Health | Denmark, Finland & Norway | Yes | No | Yes | No | Yes | No |
| Suri and Clarke 2009 (40) | Highlights methodologically inclusive advancements in research synthesis methods | Critique, opinion-based; , compares QES | Education | Australia | Yes | No | Yes | No | No | - |
| Dixon-Woods *et al. 2005 (41)* | A brief overview and critique of a selection of strategies for synthesising qualitative and quantitative evidence | Critique, empirical (literature review); compares QES | Health | UK | No | - | Yes | No | Yes | No |
| Weed 2006 (82) | Discusses a method for the interpretive  Synthesis of qualitative research: ‘‘meta-interpretation’’ | Critique, empirical | Sports science | UK | No | - | Yes | No | No | - |
| Weed 2008 (44) | Discusses the potential to develop a ‘meta-interpretation’ approach | Critique, empirical | Sports science | UK | No | - | Yes | No | No | - |
| Walsh and Downe 2005 (49) | Discusses the purpose and stages of meta-synthesis | Critique, empirical; compares QES | Health | UK | Yes | No | Yes | No | Yes | No |
| Dixon-Woods *et al.2004 (37)* | An informal review of the literature on integrating qualitative and quantitative forms of evidence | Critique, empirical; compares QES | Health | UK | No | - | No | - | No | - |
| Tong *et al.2012 (23)* | Guideline to encourage transparency in reporting syntheses of qualitative research | Reporting guideline | Health | Australia | No | - | No | - | No | - |
| Bondas and Hall 2007 (64) | To analyse the methods applied in previous meta-synthesis research and to inform researchers of epistemological and methodological issues | Reporting of methods (systematic review) | Health | Sweden & Denmark | Yes | No | No | - | No | - |
| Dixon-Woods *et al.2007 (4)* | To conduct a structured review of published reports of syntheses of qualitative research in health and healthcare | Reporting of methods (systematic review) | Health | UK | No | - | No | - | No | - |
| Hannes and Macaitis 2012 (5) | To identify newly developed methods and reveal how authors have conducted search strategies, critical appraisal , and syntheses | Reporting of methods (systematic review) | Education and labour studies | Belgium & Australia | No | - | No | - | No | - |
| France *et al 2014 (6)* | To investigate in-depth the application and reporting of methods in recent health-related meta-ethnography journal papers | Reporting of methods (systematic review) | Health | UK | Yes | No | Yes | No | Yes | No |

Key: ‘-‘ = not applicable; HTA = health technology assessment; ME = meta-ethnography with methodological detail; QES= qualitative evidence synthesis methodologies
