# Supplementary material for: A methodological systematic review of meta-ethnography conduct to articulate the complex analytical phases
Source: BMC Med Res Methodol. 2019 Feb 18;19:35. doi: 10.1186/s12874-019-0670-7 (PMC6380066; doi:10.1186/s12874-019-0670-7)
Supplement: Supplementary file 3 — Table S3. Examples of ‘rich’ and ‘not rich’ data on phases 4 to 6 from systematic review publications. A table providing data excerpts from review publications illustrating data that we judged rich and not rich on conduct of phases 4 to 6 and explaining why. (DOCX 26 kb) [file 12874_2019_670_MOESM3_ESM.docx]

**Table 3. Examples of ‘rich’ and ‘not rich’ data on phases 4 to 6 from systematic review publications**

| **Phase** | **Example extracts of ‘rich’ data** | **Our judgement** | **Examples of ‘not rich’ data** | **Our judgement** |
| --- | --- | --- | --- | --- |
| 4 | Malpass A, Shaw A, Sharp D, Walter F, Feder G, Ridd M, et al. "Medication career" or "moral career"? The two sides of managing antidepressants: a meta-ethnography of patients' experience of antidepressants. Soc Sci Med. 2009;68(1):154-68.  Example extract 1  ‘Our first step was to read and re-read the papers in chronological order to note the second order constructs. AM and AS compiled tables in which they noted the second order constructs in each paper, illustrated by raw data from the papers (first order constructs), in two separate columns. In a third column they noted additional ideas that arose as they read the papers, which fed into the development of third order constructs. The other team members compiled similar tables for the papers they had critically appraised; thus each paper was read for second order constructs by three reviewers independently. We also drew ‘conceptual maps’ for each paper to show the major second order constructs and how they related within each paper. This was in order to ‘‘preserve the structure of relationships between concepts within any given study’’ (Britten et al., 2002: 210) as the metaethnographic approach emphasizes the contextual preservation of meaning.’ (P. 158)  Example extract 2  ‘Early on in the meta-ethnography, we discerned two groups of papers with differing conceptual foci: group 1 focused on patients’ decision-making relationships with practitioners (notably GPs) and group 2 focused on the effect of antidepressants on patients’ self-concept and identity, with particular attention to stigma. As previously noted, this grouping was significant as we decided to synthesise group 1 and group 2 papers separately before drawing them together.’ (P161) | Rich in detail on conduct of meta-ethnography – explains how they did phase 4. Used as one of our illustrative case studies.  613 words of article & a table coded in Nvivo under Phase 4 | Bondas T, Hall EO. Challenges in approaching metasynthesis research. Qual Health Res. 2007;17(1):113-21.  ‘First, studies can be combined such that one study can be presented in terms of another. The accounts are then directly comparable as reciprocal translations and are analogous. Published metastudies most often seem to use this ordering, and we were surprised to discover that there is no evidence of differences between the findings of the studies (Bondas & Hall, 2006). Second, the studies can be set against one another, such that the grounds for one study’s refutation of another become visible. The accounts stand in relative opposition to each other and are essentially oppositional. Synthesizing refutations can show much about the assumptions that guided various studies by an examination of accounts, debate, and critique. The research into chronic illness by Paterson et al. (2001) penetrates this question.’ (p.118) | One paragraph only paraphrasing Noblit and Hare to describe phase 4 |
|  |  |  | McCann S, Campbell M, Entwistle V. Recruitment to clinical trials: a meta-ethnographic synthesis of studies of reasons for participation. J Health Serv Res Policy. 2013;18(4):233-41.  ‘We generated a list of key categories that served as the basis for comparing the similarities and diﬀerences across studies.’ (P. 234). | Only one sentence |
| 5 | Booth A, Carroll C, Ilott I, Low LL, Cooper K. Desperately seeking dissonance: identifying the disconfirming case in qualitative evidence synthesis. Qual Health Res. 2013;23(1):126-41.  Example extract 1  ‘Booth identified that one characteristic of systematic reviews of qualitative research is that “[p]articular attention is focused on negative or disconfirming cases. This adds to the richness of the insight that the review provides on the phenomenon of interest” (2001, p. 2). Noticeably, those reviewers using interpretative methods of synthesis—particularly those methods that trace their pedigree to grounded theory approaches—have reflected a greater preoccupation with the disconfirming case:  Grounded theory approaches also emphasise the importance of searching for negative or disconfirming cases to challenge emergent analyses . . . and this is also incorporated into the meta-study approach. . . . Similarly, as the iterative process of meta-interpretation develops, theoretical sampling seeks not only to broaden and deepen the analysis, but also to challenge it through seeking alternative points of view and perspectives. (Weed, 2007, p. 19)  Development of a wider choice of methodologies for qualitative evidence synthesis has been accompanied by increasing recognition of the value of identifying the dis- confirming case. This stems, at least in part, from a need to demonstrate review findings that go beyond the obvious and that are nuanced to particular contingencies, settings, or population subgroups.’ (p. 128)  Example extract 2  ‘Attention to analysis provides an opportunity to identify exceptions to the rule. It also allows a reviewer to explore and explain such exceptions. A review team should identify circumstances under which findings are only present in particular subgroups (cf. subgroup analysis). They should also identify whether any particular study has had a disproportionate influence on the themes present in the final synthesis (cf. sensitivity analysis; Mills, Jadad, Ross, & Wilson, 2005). Would a model or framework still be complete if the team was to remove such a study and its findings from the synthesis? Downe suggested that such testing might be formative (i.e., iterative and ongoing) through a constant comparison approach:  We have also adopted the techniques of theoretical saturation and of searching for disconfirming data, borrowed from grounded theory. As we analysed each additional study, we consciously checked if the findings extended or refuted the emerging line of argument synthesis. (2008, p. 6)’ (p. 132). | Detailed critique of refutational synthesis/translation.  Overview & review of methods for sampling deviant cases in qualitative evidence syntheses.  652 words of article coded in Nvivo under Phase 5 | Beck CT. Metasynthesis: a goldmine for evidence-based practice. Aorn J. 2009;90(5):701-2, 5-10.  ‘5. Translate the studies into one another (ie, metaphors from each study are compared with those of the other studies included in the metasynthesis). As Noblit and Hare explain,  *Translations are especially unique syntheses, because they protect the particular, respect holism, and enable comparison. An adequate translation maintains the central metaphors and/or concepts of each account in their rela- tion to other key metaphors or concepts in the account*.^8^ (p28)’ (p705) | One paragraph only citing Noblit and Hare 1988 to describe phase 5 |
|  |  |  | Systematic review publication:  Suri H, Clarke D. Advancements in Research Synthesis Methods: From a Methodologically Inclusive Perspective. Rev Educ Res. 2009;79(1):395-430.  ‘Noblit and Hare (1988) used the term metaphor to refer to “themes, perspectives, organizers, and/or concepts revealed by qualitative studies”; they recommended “metaphoric reductions” to “achieve both abstraction and complexity, and create translations that preserve the relations between concepts” (p. 14). These metaphoric reductions are then translated into one another and expressed as analogies through “idiomatic translations” of salient categories of meaning. A meta- ethnography takes varied forms depending on how individual accounts are related to one another: “as a reciprocal translation (essentially similar and subject to direct translation), as a refutation (involving translation of refutations as well as accounts), or in a line of argument (an analogy about a set of parts to some whole)” (pp. 81–82).’ (P.401) | Only part of one paragraph paraphrasing and citing Noblit and Hare 1988 |
|  |  |  | Pope C, Mays N. Synthesising qualitative research. In: Pope C, Mays N, editors. Qualitative research in health care (3rd ed). Oxford UK: Blackwell Publishing; BMJ Books; 2006. p. 142-52.  ‘A key feature of meta-ethnography is the use of *reciprocal translation* – a process in which different studies are translated or interpreted into one another. This entails systematically searching through each study, extracting key findings and interpretations, and comparing them with each other in order to develop a set of overarching concepts or overlapping areas. This process resembles the constant comparison methods used in primary qualitative research approaches such as ‘grounded theory’. Each finding (e.g. a concept or interpretation) is examined to see how it is like (or unlike) those in the other studies, and these are matched, merged and adapted to enable the generation of a new, combined set of interpretations. The product of a meta-ethnography may be simply this reciprocal translation, but more often this can be developed further into a new *‘line of argument’* synthesis.’ (p. 146-147).  ‘In general, as with the methods used in primary research, the methods for synthesis should be explicit and transparent, but the key stages should be seen as flexible, pragmatic and iterative rather than strictly sequential. Inevitably, syntheses of complex bodies of evidence, whether solely qualitative, or combining qualitative and quantitative evidence, require experience and judgement on the part of the researchers.’ (P. 150). | Two paragraphsgiving a concise summary –, useful but not rich in terms of detail on how to conduct phase 5 |
| 6 | Atkins S, Lewin S, Smith H, Engel M, Fretheim A, Volmink J. Conducting a meta-ethnography of qualitative literature: lessons learnt. BMC Med Res Methodol. 2008;8:21.  Example extract  ‘As the process of synthesising research in meta-ethnography is not clearly delineated, we agreed on a method of synthesis based on our reading of a number of existing reviews. In developing an overarching model (or third order interpretation or synthesis), we listed the translated themes and subthemes in a table, juxtaposed with secondary themes derived from author interpretations. Each member of the (multi-disciplinary) research team then independently developed an overarching model that linked together the translations and authors' interpretations. These models were then merged, discussed, and used to generate hypotheses, in order to produce a 'line- of-argument' synthesis. Each author was also asked to develop a mind map of their own model of the synthesis. Synthesising results in this manner proved rather difficult, as the interpretations of different members of the team varied widely. Inevitably, compromises needed to be made. This highlights the similarity of qualitative synthesis with primary qualitative research, in terms of the inherent subjectivity of interpretation. We also found that synthesising the large number of studies from many different contexts complicated the synthesis process.’  (P.8) | Meta-ethnography with methodological detail – described how they did phase 6.  Used as one of our illustrative case studies.  545 words of article & tables  coded in Nvivo under Phase 6 | McCormick J, Rodney P, Varcoe C. Reinterpretations across studies: an approach to meta-analysis. Qual Health Res. 2003;13(7):933-44.  ‘Synthesizing translations. Translations are the first level of a metaethnographic synthesis. The various translations can be compared with one another to determine if some metaphors and/or concepts are able to encompass those of other accounts. If so, a second level of synthesis is possible: analyzing types of competing interpretations and translating them into each other.’ (P. 939)  ‘In the final step, we created an interpretation of interpretations—a new narrative that not only accounts for the original metaphors and interpretations but also goes beyond these to describe broader cultural phenomena that were operating in all of the studies. Throughout this process, we struggled to preserve the original meanings and contexts while attempting to go beyond the individual studies to explicate the larger socioeconomic and political issues that underpin the culture of health care.’ (P.940). | Two paragraphs only - one paraphrases Noblit and Hare 1988, the other gives a very brief description of methods |
|  |  |  | Walsh D, Downe S. Meta-synthesis method for qualitative research: a literature review. J Adv Nurs. 2005;50(2):204-11.  ‘Synthesis of translation  The ﬁnal phase is synthesizing the translations to elucidate more reﬁned meanings, exploratory theories and new concepts. Clusters of metaphors become progressively more reﬁned and a consensus emerges as to core themes or explanatory, mid-level, or substantive theory (Sherwood 1997b, Strauss & Corbin 1998, Campbell et al. 2003). The synthesis needs to reﬂect the tension between contradictory or alternative explanations if reciprocal translations suggest a lack of congruence. Ultimately, the ﬁnal synthesis will be the grounds on which the value of meta-synthesis is judged and it therefore needs to convey explicitly how the whole is greater than the sum of the constituent parts.’ (p. 209) | Single paragraph – not detailed about how to conduct Phase 6 |
