# Supplementary material for: A methodological systematic review of meta-ethnography conduct to articulate the complex analytical phases
Source: BMC Med Res Methodol. 2019 Feb 18;19:35. doi: 10.1186/s12874-019-0670-7 (PMC6380066; doi:10.1186/s12874-019-0670-7)
Supplement: Supplementary file 4 — Table S4. Comparative case studies of four meta-ethnography worked examples. A table comparing how phases 4 to 6 were conducted in four ‘worked example’ review publications. (DOCX 22 kb) [file 12874_2019_670_MOESM4_ESM.docx]

**Table 4. Comparative case studies of four meta-ethnography worked examples**

| **Case study** | **Phase 4** |  |  | **Phase 5** |  |  | **Phase 6** |  |  |
| --- | --- | --- | --- | --- | --- | --- | --- | --- | --- |
| **Case study 1: Campbell et al 2011** | List & describe study concepts by hand | Organise studies into groups by focus & order chronologically within groups | Create visual ‘maps’ or diagrams to summarise key findings onto a single page for each group & draw relationships between findings |  | Within each group of studies, compare meaning of concepts study by study resulting in textual synthesis (reciprocal translations) | Create visual ‘maps’ or diagrams to summarise key findings onto a page for each group & draw relationships between findings | Compare maps across groups to produce overall model of medicine taking | Read & re-read textual translations for each group referring to original studies | Synthesise textual translations across all groups to produce overarching textual line of argument synthesis |
| **Case study 2: Atkins et al 2008** | List & describe study concepts | Thematically analyse concepts to form thematic categories |  | Order studies chronologically | Compare concepts study by study within thematic categories to translate meaning (reciprocal translation) |  | List translated themes & sub-themes & juxtapose these with authors' concepts from primary studies | Each team member independently develops overarching model to link translations & authors' concepts | Models merged, discussed & used to generate hypotheses to produce line- of-argument synthesis |
| **Case study 3: Malpass et al 2009** |  | Organise studies into (two) groups by focus |  | Identify common concepts for each group separately | Identify common concepts across the two groups | Create ‘summary definition’ (translation) for each common concept | Create visual diagrams with accompanying detailed text description to show synthesised translations for each group separately | Pull together the two syntheses into a line of argument synthesis by combining the two diagrams & descriptions into a third diagram & a table accompanied by detailed textual descriptions |  |
| **Case study 4: Toye et al 2014** | Code concepts in NVivo using a hierarchical structure.  Each team member independently interprets each concept | Compare & merge individual interpretations to form joint interpretation for each concept |  | Sort joint interpretations of concepts into conceptual categories according to common meaning .  Team members independently describe & label each category | Discuss & further interpret conceptual categories as a team using constant comparison to create further abstracted conceptual categories |  | Collaboratively develop visual structure of conceptual categories to create line of argument synthesis.  Revise diagram to develop and refine the line of argument until it expresses joint interpretation |  |  |
